# A Porcine Monoclonal Antibody Targeting a Conserved GP4 Epitope Protects against In Vivo Infection via the Induction of Broad‐Spectrum PRRSV Neutralization

**DOI:** 10.1002/advs.202508875

**Published:** 2025-09-08

**Authors:** Xu Chen, Jiakai Zhao, Pinpin Ji, Xiaoxuan Li, Hui Niu, Dian Jiao, Lu Zhang, Qianyi Zhu, Xinyu Liu, Julian A. Hiscox, James P. Stewart, Yani Sun, Qin Zhao

**Affiliations:** ^1^ College of Veterinary Medicine Northwest A&F University Yangling 712100 China; ^2^ Engineering Research Center of Efficient New Vaccines for Animals Ministry of Education Yangling 712100 China; ^3^ Key Laboratory of Ruminant Disease Prevention and Control (West) Ministry of Agriculture and Rural Affairs Yangling 712100 China; ^4^ Engineering Research Center of Efficient New Vaccines for Animals Universities of Shaanxi Province Yangling 712100 China; ^5^ Department of Infection Biology and Microbiomes Institute of Infection Veterinary and Ecological Sciences University of Liverpool Liverpool L3 5RF UK

**Keywords:** broadly neutralizing antibodies, GP4 protein, porcine antibody, PRRSV

## Abstract

Porcine reproductive and respiratory syndrome virus (PRRSV) imposes substantial economic losses on global swine production. While modified live vaccines remain the primary prevention tool, their efficacy is compromised by the genetic variability of PRRSV. This study developed a broadly neutralizing monoclonal antibody (mAb) that targets a conserved viral epitope as an alternative therapeutic strategy. Using hybridoma technology, BALB/c mice with purified PRRSV particles and screened mAb‐5F2 is immunized, which demonstrated cross‐lineage neutralization against PRRSV‐2 (lineages 1, 5, and 8) and PRRSV‐1 strains. Epitope mapping revealed two residues, Q48 and I50, of GP4, which are key motifs for interaction with mAb‐5F2. Mechanistic studies revealed that the mAb‐5F2 sterically hinders the GP4‐CD163 interaction, reducing viral entry efficiency by 78–85% at a concentration of 50 µg mL^−1^. To enhance clinical applicability, a porcine antibody (5F2‐pFc) is engineered. In vivo challenge trials revealed that 5F2‐pFc treatment completely prevented mortality in PRRSV‐infected piglets (100% survival vs 40% in controls) while significantly reducing pulmonary viral loads (2‐log decrease) and histopathological lesions. This work identified a novel conserved neutralizing epitope on PRRSV GP4 and established a porcineized antibody platform that combines broad‐spectrum neutralization with clinical practicality, offering a promising strategy to overcome current vaccine limitations in PRRSV management.

## Graphical abstract

This study developed mAb‐5F2, a monoclonal antibody targeting conserved GP4 residues (Q48/I50), which blocks the GP4‐CD163 interaction, resulting in a 78–85% reduction in viral entry. Porcineized 5F2‐pFc prevented mortality in infected piglets, reduced pulmonary viral loads, and minimized lesions. This broad‐spectrum antibody platform overcomes vaccine limitations, offering a promising therapeutic strategy for PRRSV control by combining the targeting of conserved epitopes with clinical efficacy.

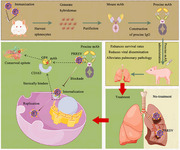

## 1 Introduction

Porcine reproductive and respiratory syndrome (PRRS), caused by the PRRS virus (PRRSV), is one of the most economically devastating diseases in swine production worldwide. In the United States alone, annual losses exceed $640 million because of reduced productivity, reproductive failure, and mortality.^[^ ^1^ ^]^ The virus exists as two genetically distinct species, *Betaarterivirus suid 1* (PRRSV‐1, European) and *Betaarterivirus suid 2* (PRRSV‐2, North American), with PRRSV‐2 exhibiting greater genetic diversity and posing greater challenges for the design of cross‐protective vaccines.^[^ ^2^ ^]^ PRRSV is a single‐stranded RNA virus with a 15.4 kb genome containing 10 open reading frames (ORFs). ORF1a and ORF1b encode nonstructural polyproteins essential for replication, and the remaining ORFs (2‐7) produce structural components. These include GP2 (ORF2), GP3 (ORF3), GP4 (ORF4), GP5 (ORF5), M protein (ORF6), and nucleocapsid protein (ORF7).^[^ ^3^ ^]^ Among these, the GP5‐M heterodimer facilitates viral attachment, whereas the GP2‐GP3‐GP4 trimer mediates subsequent entry by binding to the host receptor CD163.^[^ ^4^ ^]^ Notably, glycosylation patterns in GP5 and conformational flexibility in GP4 contribute to immune evasion, enabling persistent viral adaptation.^[^ ^5^ ^]^

Despite decades of research, preventing PRRSV infection remains a major challenge. The rapid evolution and diverse antigenic profiles of viruses limit the effectiveness of modified live vaccines (MLVs).^[^ ^6^ ^]^ MLVs mitigate clinical symptoms in homologous strains.^[^ ^7^ ^]^ However, heterologous challenge studies have demonstrated efficacy rates as low as ≈40%.^[^ ^8^ ^]^ Vaccinated herds frequently serve as reservoirs for viral recombination.^[^ ^9^ ^]^ Subunit vaccines eliminate the need for attenuated strains.^[^ ^10^ ^]^ This approach circumvents biosafety risks associated with the recombination of wild‐type strains. However, their limited cross‐protective efficacy stems from two key barriers: 1) structural proteins such as GP3/GP4 require mammalian systems for native antigenicity and posttranslational folding^[^ ^11^ ^]^ and 2) broadly conserved neutralizing epitopes remain scarce.^[^ ^12^ ^]^ To date, only seven neutralizing epitopes have been identified across PRRSV structural proteins (GP2, GP3, GP4, GP5, and M), all of which exhibit strain‐specific activity.^[^ ^13^ ^]^ For example, the GP5 epitope (aa 31–50) triggers neutralizing antibodies in 60% of vaccinated pigs but fails to block lineage 8 strains prevalent in Asia.^[^ ^14^ ^]^ Even the broadly reactive mAb PR5nf1, which targets an undefined, conserved epitope, has limited clinical value due to its IgM isotype.^[^ ^15^ ^]^ Therefore, to develop highly effective PRRSV subunit vaccines, broadly conserved neutralizing epitopes remain to be screened and identified.

Additionally, in recent years, the development of antiviral drugs has offered another avenue for preventing PRRSV. However, some small‐molecule compounds and herbal extracts have been shown to inhibit PRRSV replication in vitro effectively but exhibit limited efficacy in vivo.^[^ ^16^ ^]^ Antibody‐based therapeutics have emerged as promising antiviral agents against PRRSV.^[^ ^17^ ^]^ However, their broad application is hindered by limited cross‐strain efficacy and concerns about immunogenicity. Screening broadly neutralizing antibodies (bnAbs) and engineering bnAbs may partially address these limitations.

Furthermore, identifying conserved epitopes targeted by bnAbs could provide a structural blueprint for designing subunit vaccines with broader protection. In this study, BALB/c mice were immunized with intact PRRSV particles to generate bnAbs. Epitope mapping revealed that a bnAb targets a highly conserved region of the PRRSV GP4 protein, effectively blocking viral entry by disrupting the GP4‐CD163 interaction. To enhance therapeutic potential, the murine IgG Fc domain was replaced with a porcine IgG Fc fragment through engineering. The engineered swine‐adapted bnAb potently inhibited PRRSV infection in pigs. These findings advance our understanding of PRRSV antigenicity and provide promising therapeutic candidates for combating PRRSV infections.

## 2 Result

### 2.1 PRRSV Particles Induce Robust Immune Responses in Mice

**Figure 1:**
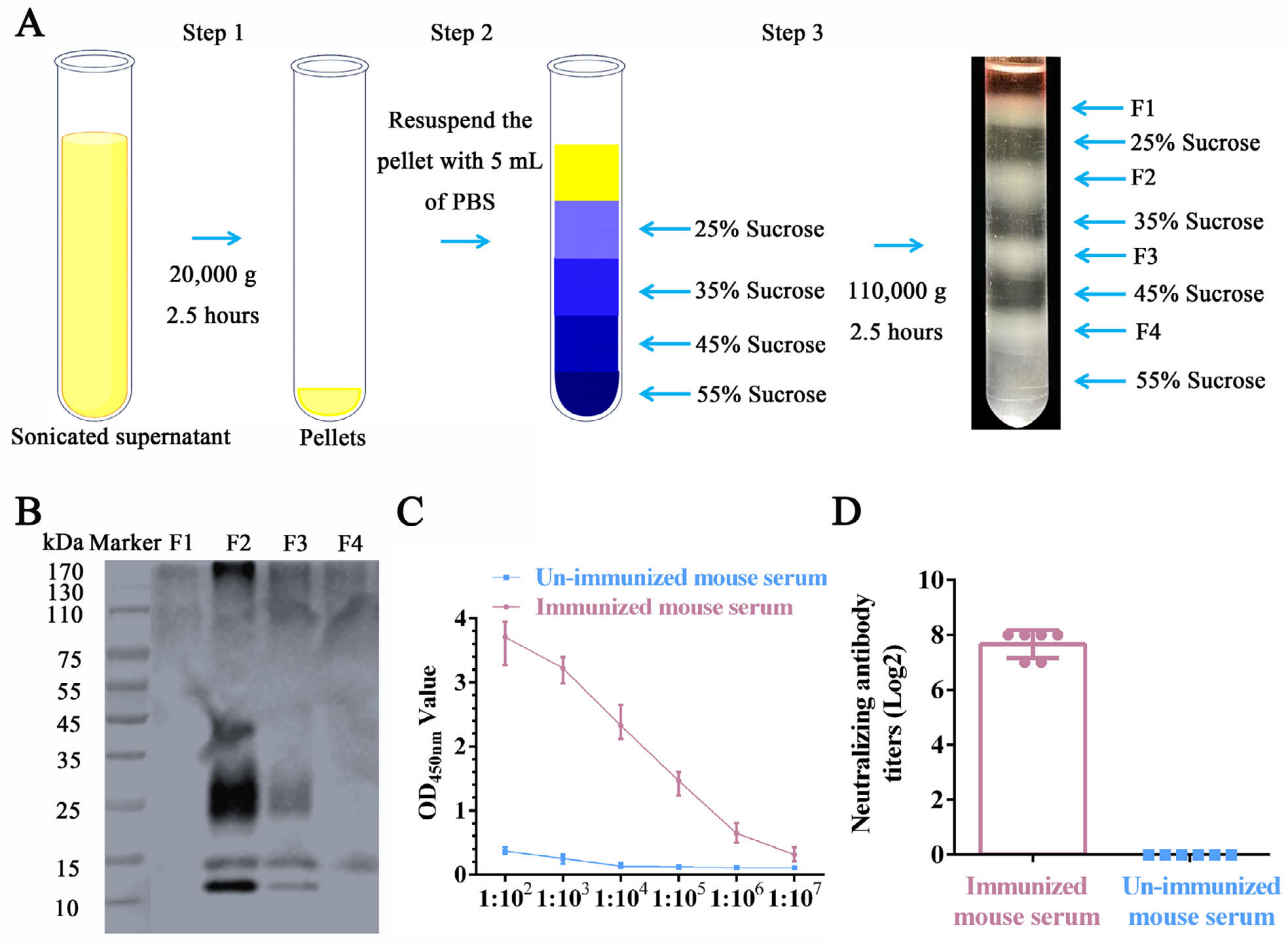
PRRSV particles induce robust immune responses in BALB/c mice. A) Purification of PRRSV particles via sucrose concentration gradient centrifugation. B) Western blotting analysis of the purified PRRSV particles. Pig sera were positive for PRRSV as the primary antibody. C) Titers of antibodies against PRRSV in the immunized mice by indirect ELISA. D) Neutralizing titers of the immunized mice were determined via a virus neutralization assay. F1: Less than the 25% sucrose interface, F2: 25–35% sucrose interface, F3: 35–45% sucrose interface, and F4: 45–55% sucrose interface. The symbols represent three independent biological replicates, and the bar graph shows the means ± SEM values of the samples, with *P* values as indicated, which were calculated using Student's two‐tailed *t*‐test.

Following centrifugation of the PRRSV stock through a sucrose density gradient, distinct protein bands were observed in the 0–25%, 25–35%, 35–45% and 45–55% sucrose ranges (**Figure** **1**A). Western blotting analysis revealed that the 25%‐35% sucrose gradient contained the highest concentration of purified PRRSV particles (Figure 1B).

BALB/c mice were immunized with purified PRRSV particles. One week after the fourth immunization, indirect ELISA revealed that the serum antibody titers against PRRSV ranged from 1:100000 to 1:1000000 (Figure 1C), indicating the induction of strong humoral immunity. Subsequent virus neutralization assays using these sera revealed neutralizing titers of 1:128–1:256 (Figure 1D), confirming the functional activity of the elicited antibodies.

### 2.2 Monoclonal Antibody Generation Against PRRSV Particles

**Figure 2:**
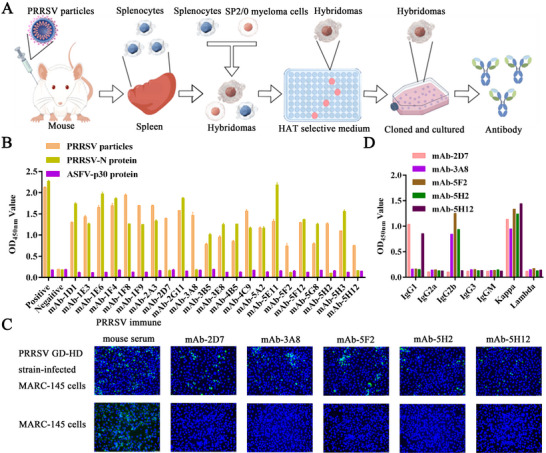
Preparation of monoclonal antibodies against PRRSV. A) Schematic diagram of hybridoma cell preparation. B) Screening of anti‐PRRSV monoclonal antibodies via indirect ELISA. C) Identification of monoclonal antibodies that bind to PRRSV via IFA. MARC‐145 cells were infected with or without PRRSV (GD‐HD strain). Representative pictures were taken with a scale bar of 100 µm. D) Identification of subtypes of monoclonal antibodies via indirect ELISA. The symbols represent three independent biological replicates. The data were collected and analyzed via Microsoft Excel (Excel 2016) and visualized via GraphPad Prism.

Splenocytes isolated from BALB/c mice immunized with PRRSV particles were fused with SP2/0 myeloma cells via PEG‐mediated fusion. Hybridoma clones were selected using HAT medium and limiting dilution cloning according to standard protocols (**Figure** **2**A).^[^ ^18^ ^]^ For antigen specificity screening, two indirect ELISAs were performed separately using purified PRRSV particles and the PRRSV N protein as coating antigens. Among the 22 hybridoma supernatants that were reactive against PRRSV particles, 17 also cross‐reacted with the N protein, suggesting that the remaining five clones recognized other structural proteins (Figure 2B). To eliminate the reaction with nonspecific antigens, PRRSV‐infected and PRRSV‐uninfected monkey embry kidney epithelial cells (MARC145 cells) were validated by IFA analysis. These five supernatants bound to PRRSV GD‐HD strain‐infected MARC‐145 cells and did not bind to MARC‐145 cells (Figure 2C), indicating that recognition of non‐N protein epitopes potentially targeted the GP2, GP3, GP4, GP5, or M proteins specifically (Figure 2C).

Five candidate mAbs (mAb‐5F2, mAb‐5H2, mAb‐5H12, mAb‐2D7, and mAb‐3A8) were obtained from the supernatant of hybridoma cells and purified via protein G affinity chromatography. Subtype analysis via commercial kits revealed that mAb‐5F2, mAb‐5H2, and mAb‐3A8 belong to the IgG2b isotype with κ light chains, whereas mAb‐5H12 and mAb‐2D7 were classified as IgG1 κ (Figure 2D).

### 2.3 Screening of Neutralizing Monoclonal Antibodies Against PRRSV

**Figure 3:**
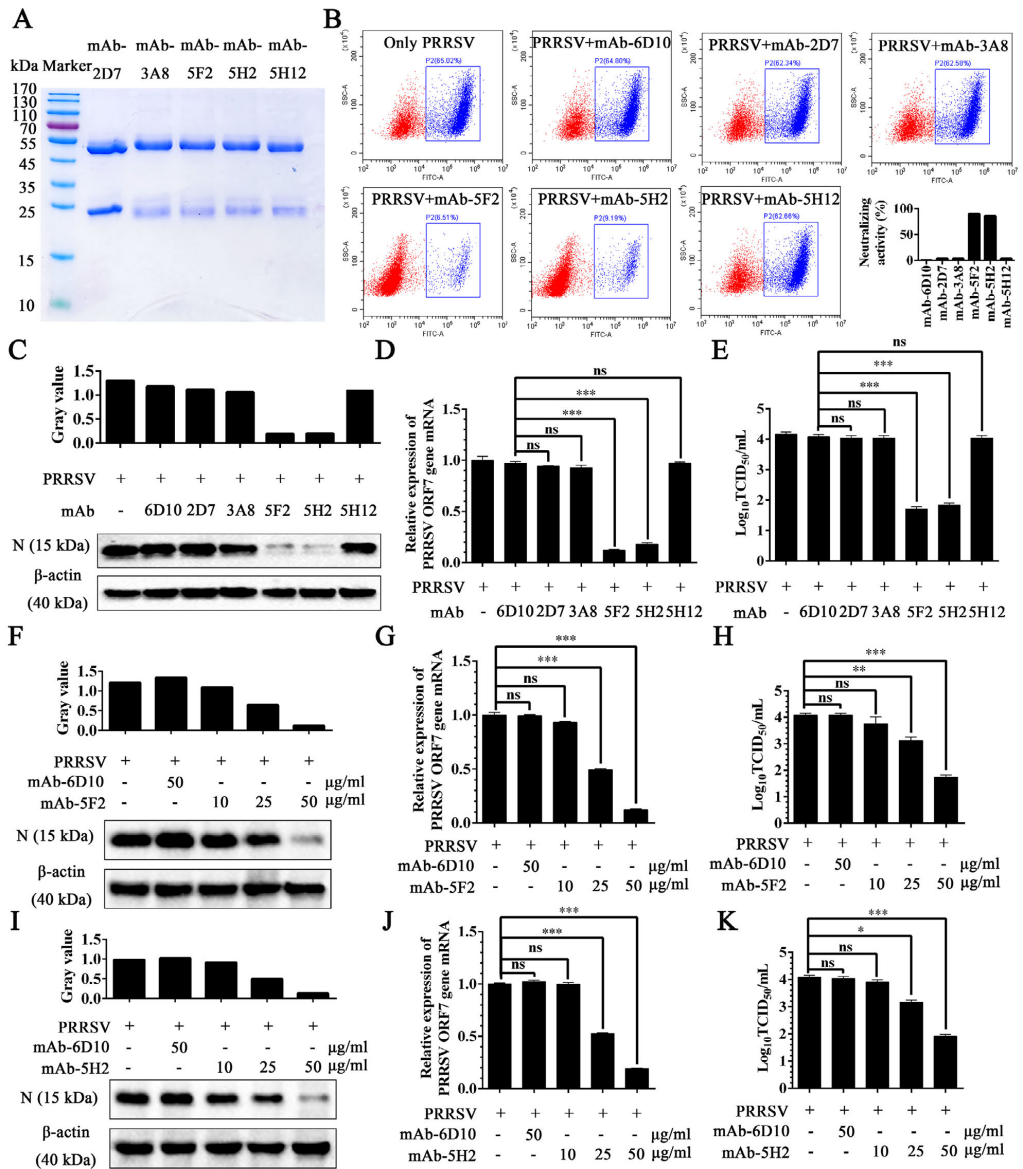
Screening of neutralizing monoclonal antibodies against PRRSV. A) SDS‐PAGE analysis of the purified monoclonal antibodies. B) Screening of neutralizing monoclonal antibodies was performed via flow cytometry. C) The neutralization effects of different monoclonal antibodies were evaluated by western blotting to analyze PRRSV N protein levels in PAM cells. D) RT‐qPCR detection of ORF7 mRNA. E) The TCID_50_ of progeny viruses in the supernatant. F) The dose‐dependent effect of mAb‐5F2 and I) mAb‐5F2 were evaluated via western blotting analysis of PRRSV N protein levels in PAM cells. G) and J) RT‐qPCR detection of PRRSV ORF7 mRNA. H) and K) The TCID_50_ of progeny viruses in the cell supernatant. The symbols represent three independent biological replicates. The data are presented as the means ± SD from a representative experiment. The data were collected and analyzed via Microsoft Excel (Excel 2016) and visualized via GraphPad Prism. *p* values were calculated using one‐way analysis of variance (ANOVA), with Dunnett's or Tukey's multiple comparisons post hoc tests employed to assess statistical significance. Statistical significance is indicated as follows: *p* < 0.05 (^∗^), *p* < 0.01 (^∗∗^), *p* < 0.001 (^∗∗∗^), and “ns” denotes no significant difference.

The 5 purified mAbs were analyzed by SDS‐PAGE, which revealed only light (25 kDa) and heavy (55 kDa) chains, as expected (**Figure** **3**A). To evaluate the neutralization capacity, procine alveolar macrophages (PAM cells) were infected with 0.1 MOI of GFP‐PRRSV (upon infection of PAM cells with GFP‐PRRSV, green fluorescence appeared at the PAM cells).^[^ ^19^ ^]^ Flow cytometric analysis demonstrated that mAb‐5F2 and mAb‐5H2 achieved significant neutralization rates of 89.98% and 85.87%, respectively, at a concentration of 50 µg mL^−1^ (Figure 3B). When tested against the GD‐HD strain (lineage 8, 0.1 MOI), both mAbs exhibited potent neutralization activity in PAMs (Figure 3C–E). Western blotting analysis confirmed reduced PRRSV N protein expression (Figure 3C), and RT‐qPCR revealed decreased ORF7 mRNA levels (Figure 3D) in mAb‐treated cells compared with those in PRRSV‐only and isotype‐matched control mAb‐6D10 cells. Moreover, TCID_50_ quantification revealed a 2‐log reduction in the number of viral progeny from the mAb‐5F2/5H2‐treated cultures at 24 h postinfection (hpi) (Figure 3E).

Dose‐dependent neutralization was further demonstrated for the mAbs 5F2 and 5H2. At 25 µg mL^−1^, both mAbs significantly inhibited GD‐HD strain replication in PAMs, whereas 10 µg mL^−1^ did not have comparable effects (Figure 3F–K). These results establish mAb‐5F2 and mAb‐5H2 as GD‐HD strain‐neutralizing antibodies with dose‐dependent activity.

### 2.4 Neutralizing Activity of mAb‐5F2 Against Diverse PRRSV Strains

**Figure 4:**
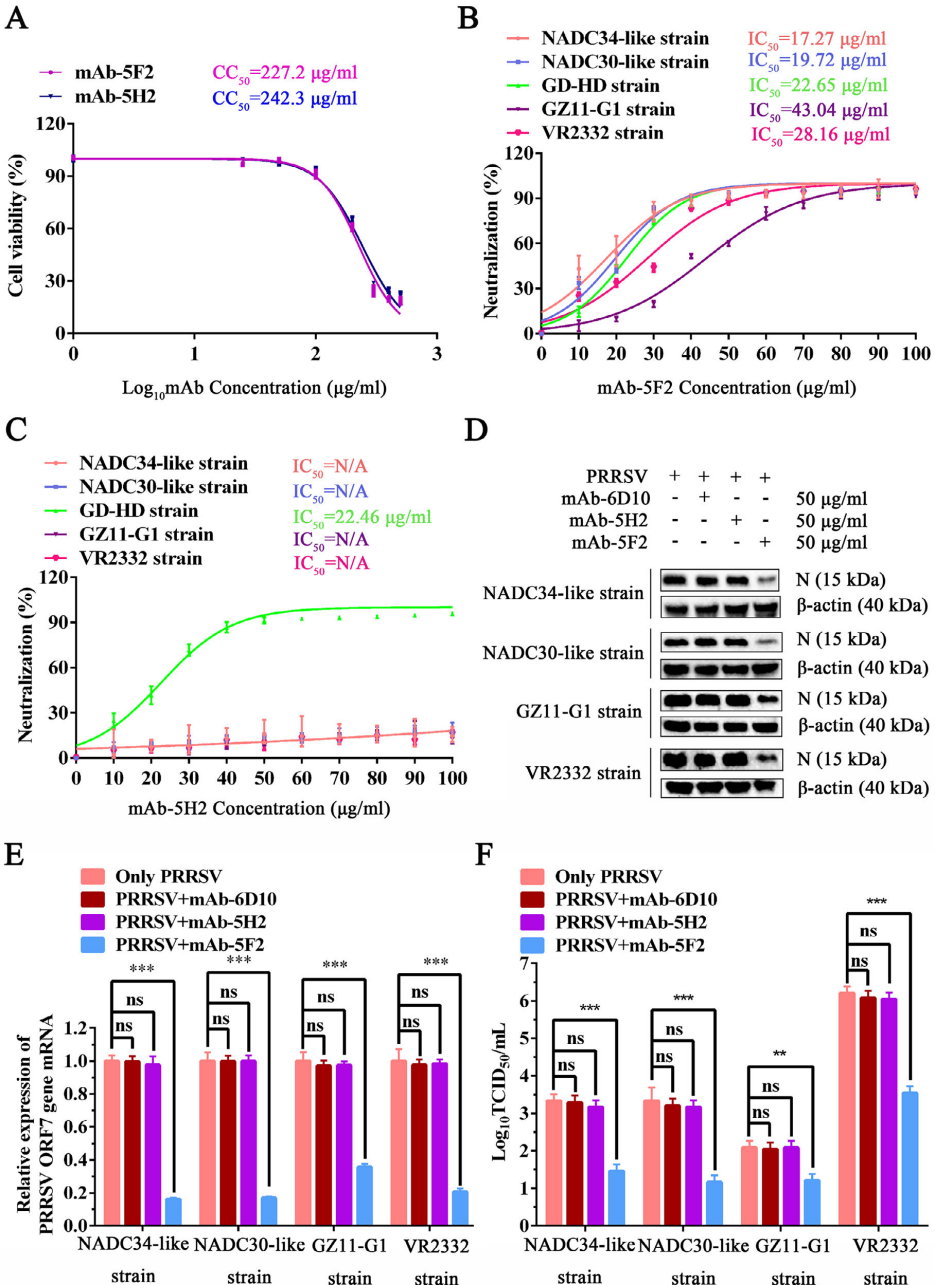
Neutralizing activity of mAb‐5F2 against diverse PRRSV strains. A) The CC_50_ values of mAb‐5F2 and mAb‐5H2 were determined via a CCK‐8 assay. B) The IC_50_ values of mAb‐5F2 against the NADC34‐like, NADC30‐like, GD‐HD, GZ11‐G1, and VR2332 strains were determined via immunofluorescence detection of the PRRSV N protein. C) IC_50_ values of mAb‐5H2 against the NADC34‐like, NADC30‐like, GD‐HD, GZ11‐G1 and VR2332 strains. D) The neutralizing activities of mAb‐5F2 and mAb‐5H2 were evaluated via western blotting analysis of PRRSV N protein levels, E) ORF7 mRNA levels, and F) the progeny virus titer. The symbols represent three independent biological replicates. The data are presented as the means ± SD from a representative experiment. The data were collected and analyzed via Microsoft Excel (Excel 2016) and visualized via GraphPad Prism. *p* values were calculated using one‐way analysis of variance (ANOVA), with Dunnett's or Tukey's multiple comparisons post hoc tests employed to assess statistical significance. Statistical significance is indicated as follows: *p* < 0.05 (^∗^), *p* < 0.01 (^∗∗^), *p* < 0.001 (^∗∗∗^), and “ns” denotes no significant difference.

To evaluate the breadth of neutralization and determine that the anti‐PRRSV activities of mAb‐5F2 and mAb‐5H2 are not attributable to their cytotoxic effects, 50% cytotoxic concentration (CC_50_) values were calculated. The CC_50_ values of mAb‐5F2 and mAb‐5H2 were 227.2 and 242.3 µg mL^−1^ in PAMs, respectively (**Figure** **4**A). We subsequently assessed the effects of mAb‐5F2 and mAb‐5H2 against multiple PRRSV strains, including GD‐HD (lineage 8), NADC34‐like/NADC30‐like (lineage 1), VR2332 (lineage 5), and GZ11‐G1 (genotype 1 PRRSV). As shown in Figure 4B,C, mAb‐5F2 had an IC_50_ (50% inhibition concentration) value of 22.65 µg mL^−1^ against the GD‐HD strain, whereas mAb‐5H2 displayed comparable activity, with an IC_50_ of 22.46 µg mL^−1^. Notably, mAb‐5F2 also effectively neutralized other strains: 17.27 µg mL^−1^ (NADC34‐like strain), 19.72 µg mL^−1^ (NADC30‐like strain), 28.16 µg mL^−1^ (VR2332 strain), and 43.04 µg mL^−1^ (GZ11‐G1 strain). In contrast, mAb‐5H2 failed to demonstrate measurable neutralization activity against these strains (Figure 4B,C). mAb‐5H2 exhibited strain‐specific neutralization, which inhibited only the replication of the GD‐HD strain under identical experimental conditions. However, mAb‐5F2 showed broad‐spectrum neutralizing effect against different PRRSV isolates.

Functional validation confirmed that mAb‐5F2, but not mAb‐5H2, potently inhibited PRRSV replication across all the tested strains in PAM cells. Compared with isotype‐matched control mAb‐6D10 treatment, mAb‐5F2 (50 µg mL^−1^) treatment resulted in significantly lower PRRSV N protein levels (Figure 4D), ORF7 mRNA copy numbers (Figure 4E), and viral progeny titers (Figure 4F) across all strain groups. Conversely, these findings collectively establish mAb‐5F2 as a broadly neutralizing antibody with potent cross‐strain activity.

### 2.5 Inhibition of PRRSV Internalization by mAb‐5F2

**Figure 5:**
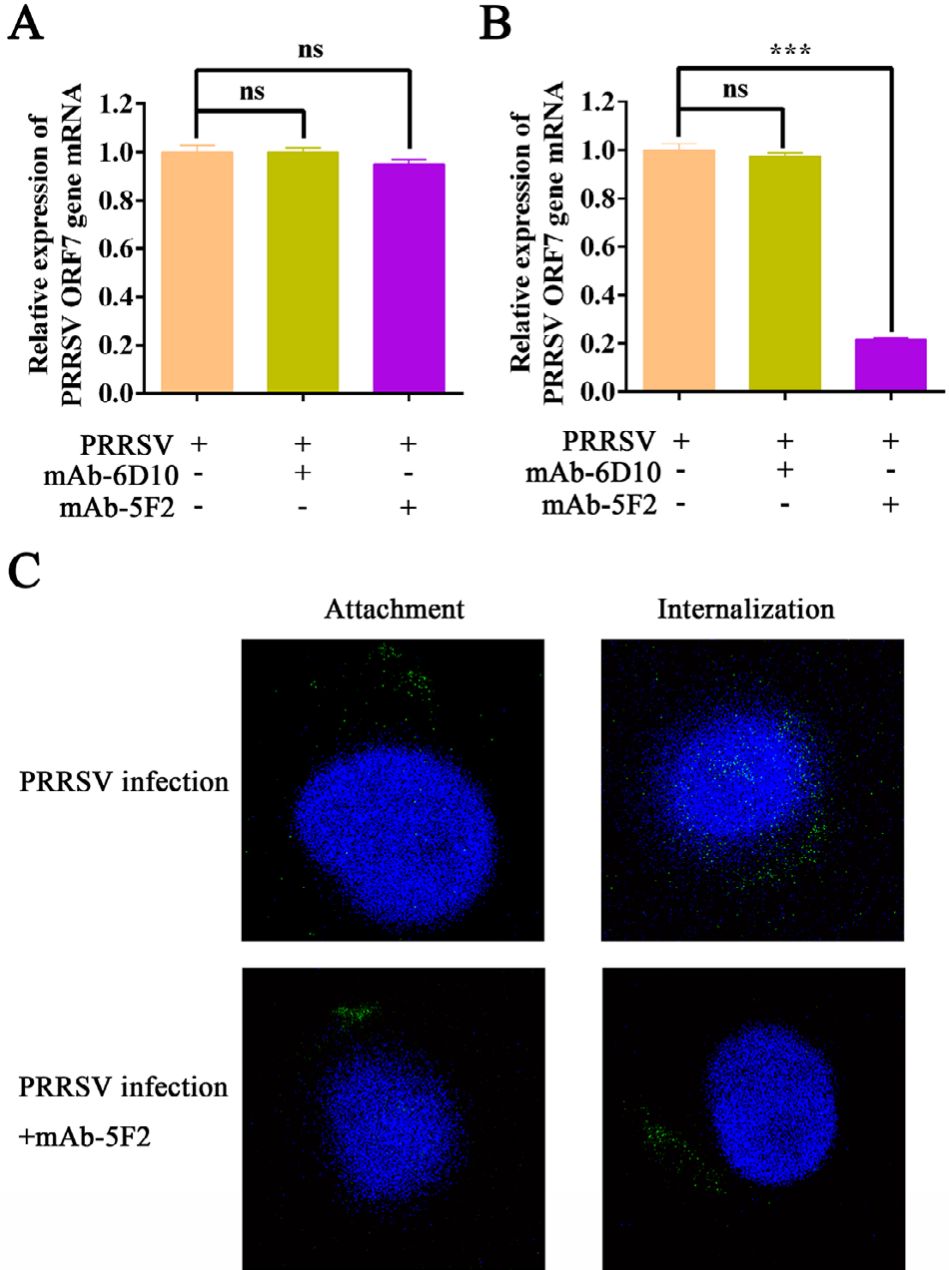
Inhibition of PRRSV internalization by mAb‐5F2. A) mAb‐5F2 did not affect virus attachment, as determined by RT‐qPCR. B) mAb‐5F2 can affect virus internalization, as shown by RT‐qPCR. C) Confocal microscopy analysis revealed that mAb‐5F2 can affect virus internalization. Representative pictures were taken with a scale bar of 10 µm. The symbols represent three independent biological replicates. The data are presented as the means ± SD from a representative experiment. The data were collected and analyzed via Microsoft Excel (Excel 2016) and visualized via GraphPad Prism. *p*‐values were evaluated using Student's two‐tailed t‐test. Statistical significance is indicated as follows: *p* < 0.05 (^∗^), *p* < 0.01 (^∗∗^), *p* < 0.001 (^∗∗∗^), and “ns” denotes no significant difference.

To determine the replication stage targeted by mAb‐5F2, we performed attachment and internalization assays. Viral RNA levels were quantified via RT‐qPCR to assess both stages. The results revealed no significant differences in PRRSV ORF7 mRNA levels between the mAb‐6D10 control group and the mAb‐5F2‐treated group during the attachment phase (**Figure** **5**A). However, marked differences were observed during internalization (Figure 5B). Confocal microscopy analysis revealed a significant reduction in intracellular PRRSV fluorescence after treatment with 100 µg mL^−1^ mAb‐5F2 (Figure 5C), indicating efficient inhibition of virus internalization. These findings collectively suggest that mAb‐5F2 primarily targets the internalization stage of PRRSV replication.

### 2.6 Fine Identification of the Antigenic Epitope Recognized by mAb‐5F2

**Figure 6:**
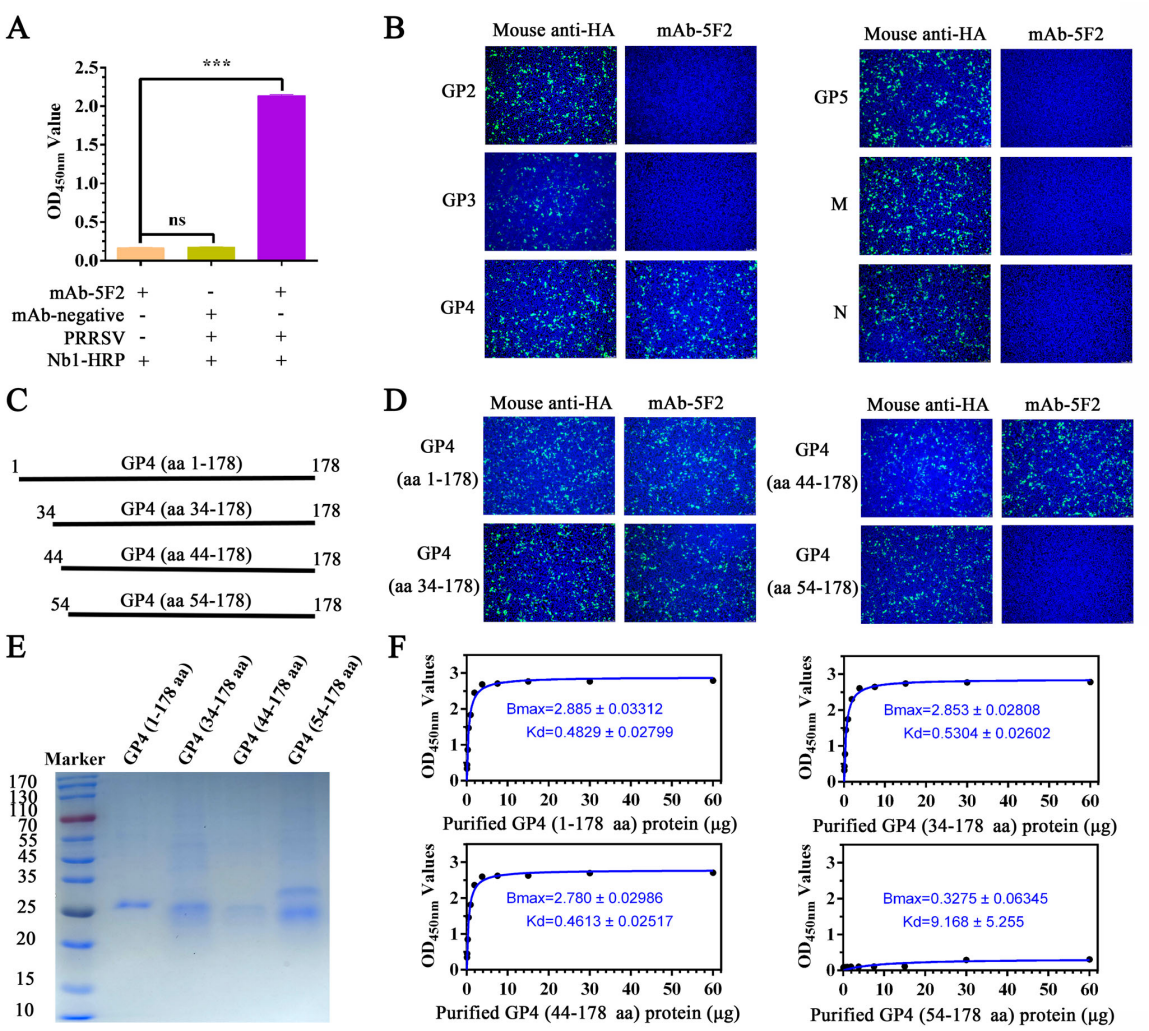
Fine identification of the antigenic epitope recognized by mAb‐5F2. A) A sandwich ELISA was performed to determine whether mAb‐5F2 captured PRRSV particles. B) The specific target proteins recognized by mAb‐5F2 were evaluated by IFA. Different PRRSV structural proteins, including GP2, GP3, GP4, GP5, M, and N, were expressed in HEK293T cells. Representative pictures were taken with a scale bar of 100 µm. C) Schematic diagram of different truncated GP4 proteins, including GP4 (aa 34–178), GP4 (aa 44–178), and GP4 (aa 54–178). D) Epitope mapping of the GP4 protein was evaluated by IFA. Representative pictures were taken with a scale bar of 100 µm. E) SDS‐PAGE analysis of GP4 and its three truncated GP4 proteins. F) Affinity assessment identified the key amino acid region recognized by mAb‐5F2 as amino acids 44–54, with Bmax denoting affinity. The symbols represent three independent biological replicates. The data are presented as the means ± SD from a representative experiment. The data were collected and analyzed via Microsoft Excel (Excel 2016) and visualized via GraphPad Prism. *p*‐values were evaluated using Student's two‐tailed t‐test. Statistical significance is indicated as follows: *p* < 0.05 (^∗^), *p* < 0.01 (^∗∗^), *p* < 0.001 (^∗∗∗^), and “ns” denotes no significant difference.

**Figure 7:**
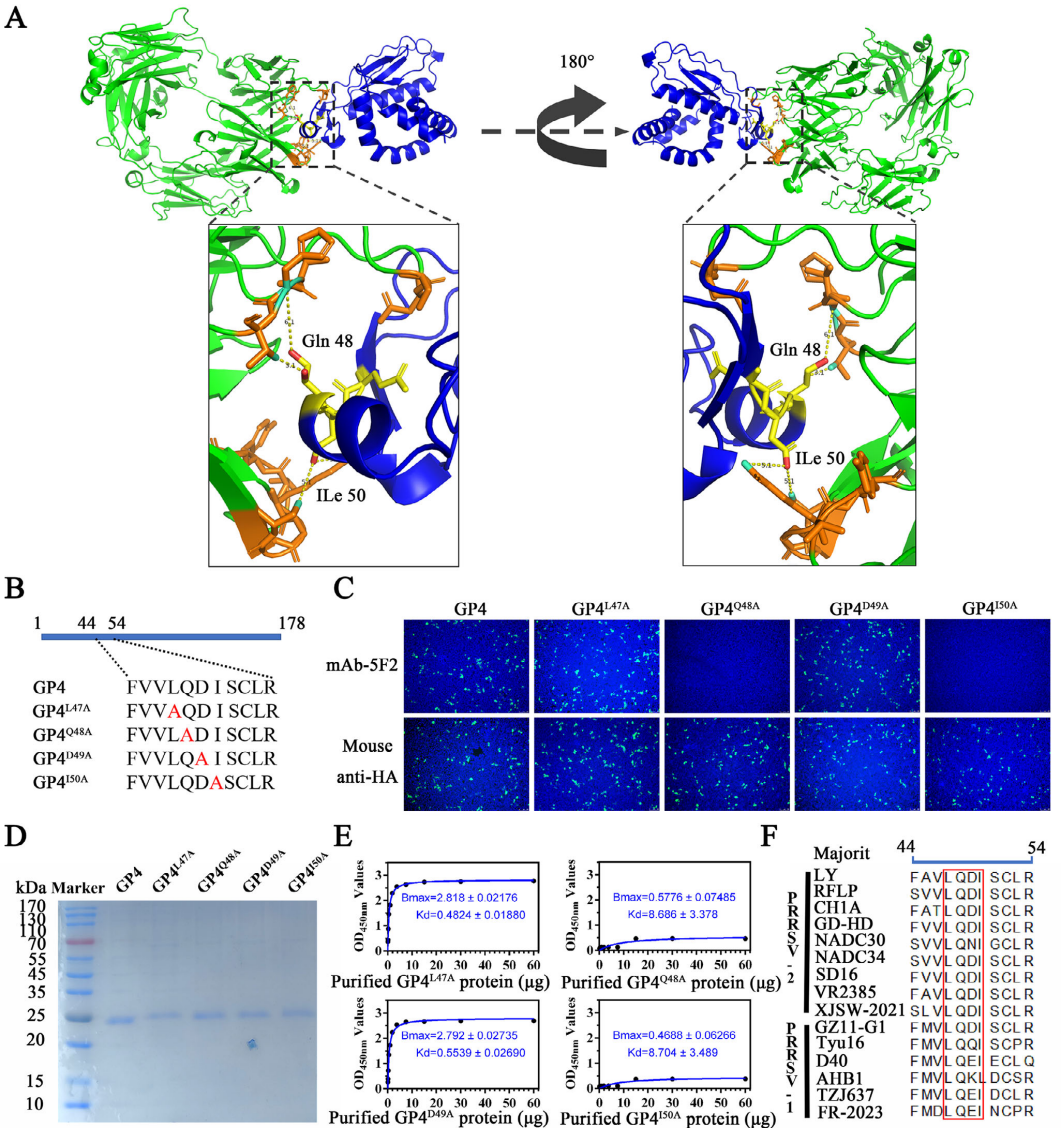
Two amino acids, Q48 on the α helix and I50 on the loop, are the key motifs for the interaction of mAb‐5F2 with GP4. A) The 3D structure of GP4 and its molecular docking with mAb‐5F2 were predicted via SwissModel and Discovery Studio Client software. B) Schematic diagram of the residue mutation of GP4. C) The key motifs involved in the interaction of mAb‐5F2 with GP4 were evaluated via IFA. Representative pictures were taken with a scale bar of 100 µm. D) SDS‐PAGE analysis of recombinant GP4 and its four mutant GP4 proteins. E) Affinity assessment revealed that the key amino acids Q48 and I50 of GP4 were recognized by mAb‐5F2, with Bmax denoting affinity. F) Amino acid sequence alignments of amino acids L47, Q48, D49, and I50 of GP4 from different PRRSV‐1 and ‐2 isolates. The symbols represent three independent biological replicates. The data were collected and analyzed via Microsoft Excel (Excel 2016) and visualized via GraphPad Prism.

To investigate the broad‐spectrum neutralization mechanism of mAb‐5F2, we first performed epitope mapping via a virion capture assay. As shown in **Figure** **6**A, mAb‐5F2 was used as the capture antibody in a sandwich ELISA format, with nanobody Nb1‐HRP serving as the detection antibody.^[^ ^20^ ^]^ Moreover, we performed western blotting with purified PRRSV using mAb‐5F2 as the primary antibody; however, no clear reaction band was detected (data not shown). The results demonstrated efficient virion capture by mAb‐5F2, suggesting that it interacts with PRRSV structural proteins and recognizes a conformational epitope (Figure 6A). To identify specific target proteins, we expressed PRRSV structural proteins (GP2, GP3, GP4, GP5, M, and N) in HEK293T cells. IFA analysis revealed that mAb‐5F2 specifically recognized GP4 (Figure 6B). For fine conformational epitope mapping, three truncated GP4 fragments encompassing the CD163‐binding domain were generated (Figure 6C),^[^ ^21^ ^]^ which included GP4 (aa 34–178), GP4 (aa 44–178), and GP4 (aa 54–178). The IFA results revealed that both GP4 (aa 34–178) and GP4 (aa 44–178) reacted with mAb‐5F2, whereas GP4 (aa 54–178) did not (Figure 6D). Moreover, the kinetics of the binding of mAb‐5F2 to GP4 and its truncated fragments were assessed via ELISA. When different amounts of GP4 and its protein truncated fragments were used as the coating antigen, the K_d_ values of mAb‐5F2 for GP4, GP4 (aa 34–178), GP4 (aa 44–178), and GP4 (aa 54–178) were 0.4829 ± 0.02799, 0.5304 ± 0.02602, 0.4613 ± 0.02517, and 9.168 ± 5.255, respectively (Figure 6F). The Bmax values of the binding capacity were 2.885 ± 0.03312, 2.853 ± 0.02808, 2.780 ± 0.02986, and 0.3275 ± 0.06345, respectively (Figure 6F). mAb‐5F2 showed significantly reduced binding affinity for GP4 (aa 54–178). These findings indicated that the neutralizing epitope resides within the aa 44–54 region of GP4. To determine the key amino acids involved in binding, we constructed the 3D structure of GP4 and performed molecular docking with mAb‐5F2 via the Swiss Model and Discovery Studio Client software. The docking model revealed that residues Q48 on the α helix and I50 on the loop of GP4 are critical for antibody recognition (**Figure** **7**A). To precisely identify the key amino acid sites of GP4 and mAb‐5F2 binding. Four mutant GP4 proteins (GP4^L47A^, GP4^Q48A^, GP4^D49A^ and GP4^I50A^) were successfully expressed in HEK293T. Mutagenesis analysis confirmed that alanine substitutions at Q48 and I50 (GP4^Q48A^ and GP4^I50A^) abolished mAb‐5F2 binding (Figure 7B,C). Then, four mutant GP4 proteins were successfully purified by Ni‐Resin with a molecular weight of ≈25 kDa (Figure 7D). ELISA quantification revealed that the dissociation constants (Bmax values) of these four mutants with mAb‐5F2 were 2.818 ± 0.02176, 0.5776 ± 0.07485, 2.792 ± 0.02735, and 0.4688 ± 0.06266, respectively (Figure 7E). The results indicate that the affinity of GP4^Q48A^ and GP4^I50A^ for mAb‐5F2 is significantly decreased, and Q48 and I50, which are located in the 44–54 amino acid region of the GP4 protein, are key amino acids for mAb‐5F2 binding. Furthermore, sequence alignment of GP4 proteins from 15 PRRSV strains, performed using the DNAStar alignment program, revealed that Q48 and I50 are highly conserved across different lineages (Figure 7F). These findings collectively establish that mAb‐5F2 targets two conserved key residues, namely, Q48 and I50, of GP4, conferring broad‐spectrum neutralization activity against diverse PRRSV isolates.

### 2.7 Blockade of GP4‐CD163 Receptor Binding by mAb‐5F2

**Figure 8:**
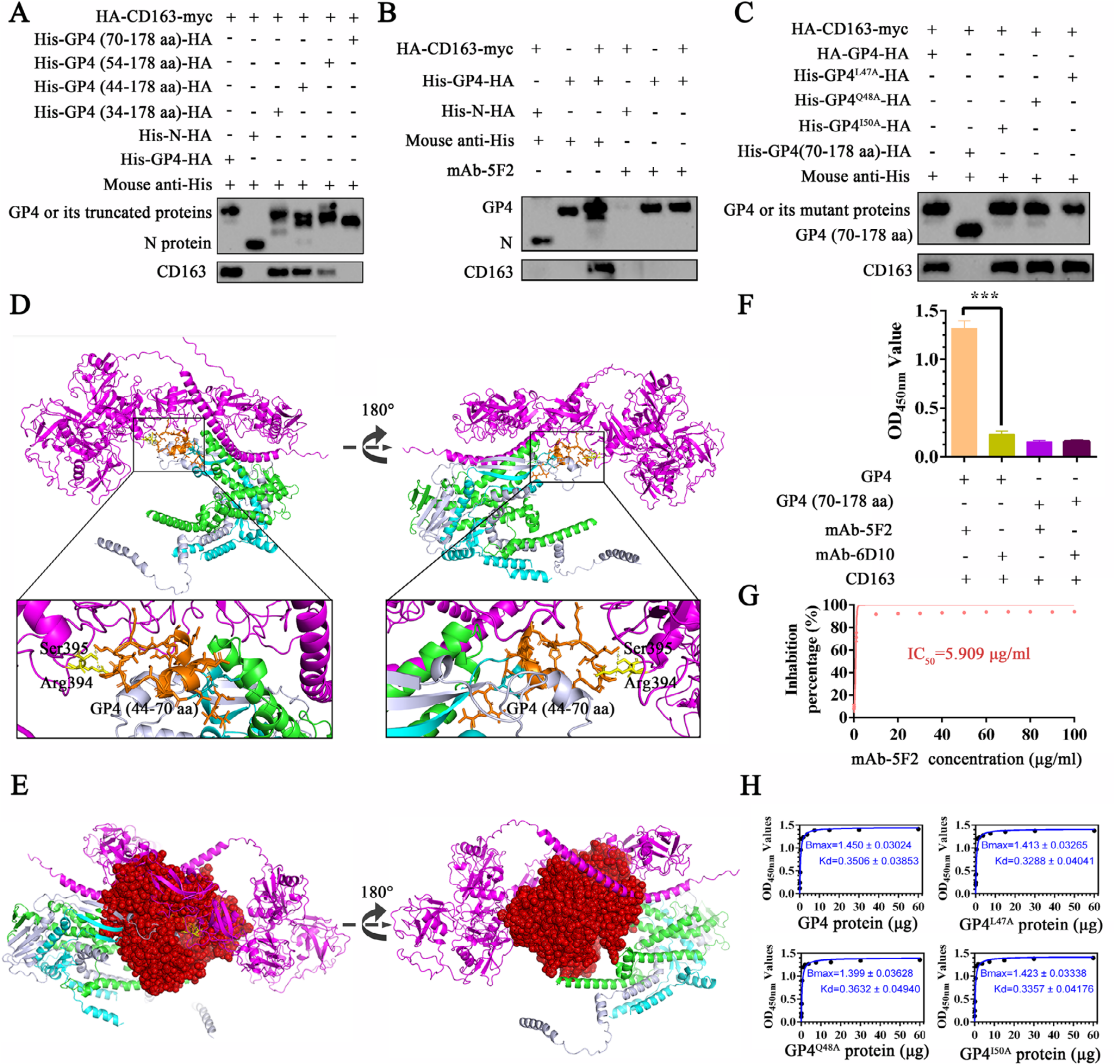
Blockade of GP4‐CD163 receptor binding by mAb‐5F2. A) The interaction region between GP4 and CD163 was identified by Co‐IP. B) mAb‐5F2 blocked the binding of GP4 to CD163. The CD163 protein was not pulled down via mAb‐5F2 as bait to first bind to GP4 in a pull‐down assay in HEK293T cells separately transfected with the recombinant pCAGEN‐HA‐CD163‐myc and pCAGEN‐His‐GP4‐HA plasmids. C) mAb‐5F2 recognized Q48 and I50 on GP4, form steric hindrance to block the binding of GP4 to CD163. D) 3D structure of the GP2‐GP3‐GP4 trimer and its molecular docking with CD163. E) The 3D structure shows steric blockade of the GP4‐CD163 receptor after GP4 bound to mAb‐5F2. PRRSV GP4 is shown in cyan, and the key amino acids are shown in orange. The CD163 receptor is shown in magenta, and the key amino acids are shown in chat. The binding of mAb‐5F2 to GP4 is shown in yellow. GP2 is shown in green. GP3 is shown in white. F) The binding of GP4 to CD163 was blocked by mAb‐5F2, as determined by blocking ELISA. G) mAb‐5F2 blocks the binding of GP4 to CD163. H) Affinity analysis of GP4^Q48A^ and GP4^I50A^ with CD163. The symbols represent three independent biological replicates. The data are presented as the means ± SD from a representative experiment. The data were collected and analyzed via Microsoft Excel (Excel 2016) and visualized via GraphPad Prism. *p*‐values were evaluated using Student's two‐tailed *t*‐test. Statistical significance is indicated as follows: *p* < 0.05 (^∗^), *p* < 0.01 (^∗∗^), *p* < 0.001 (^∗∗∗^), and “ns” denotes no significant difference.

Previous studies have demonstrated that CD163 functions as a crucial receptor for PRRSV attachment and internalization in host cells, with the GP2‐GP3‐GP4 trimer mediating receptor interaction.^[^ ^21^ ^]^ Another study identified a high‐affinity peptide, WHE, to GP4 obtained via the phage biopanning method, which could prevent PRRSV penetration and has a structure similar to the initial part of the SRCR5 domain of CD163.^[^ ^22^ ^]^ The binding region between the candidate peptide and the PRRSV GP4 protein was subsequently predicted via the template‐based protein‐protein complex structure prediction tool Spring Online, which indicated that the binding site was located mainly within the aa 23–50 region of GP4.^[^ ^22^ ^]^ Therefore, based on these previous results, we cotransfected different truncated forms of the GP4 protein with CD163 into HEK293T cells. The results of Co‐IP demonstrated that the truncated forms of GP4, including GP4 (aa 34–178) and GP4 (aa 44–178), retained the capacity to bind CD163 (**Figure** **8**A). In contrast, GP4 (aa 70–178) failed to interact with CD163, and the interaction of GP4 (aa 54–178) with CD163 was weakened, identifying the key motif of GP4 (aa 44–70) between GP4 and CD163 (Figure 8A).

To investigate whether mAb‐5F2 disrupts GP4‐CD163 binding, we performed two binding assays with the recombinant proteins. As shown in Figure 8B, GP4 and CD163 were individually expressed in HEK293T cells. GP4 failed to interact with CD163 when mAb‐5F2 was used as the primary antibody, whereas robust binding was observed when a mouse anti‐His tag antibody was used, indicating that mAb‐5F2 is capable of blocking the interaction between GP4 and CD163. It was concluded that mAb‐5F2 blocks GP4/CD163 binding, either by competitive binding to key amino acid sites or through steric effects. Furthermore, the interaction between mutated GP4 and CD163 was also determined. The results of Co‐IP revealed that the GP4^Q48A^ and GP4^I50A^ mutants retained their CD163‐binding capacity (Figure 8C), suggesting that the amino acids Q48 and I50 may not be key motifs involved in the interaction between GP4 and CD163. These results indicate that mAb‐5F2 may prevent the binding of GP4 to CD163 through steric hindrance (Figure 8B,C). A model of the interaction between the GP2‐GP3‐GP4 trimer and CD163 was also constructed. The docking model revealed that the R394 and S395 residues in the SRCR5 domain of CD163 specifically interact with the residues at positions 44–70 of GP4 (Figure 8D). By occupying the key binding interface of this domain, mAb‐5F2 has a steric hindrance effect, thereby effectively inhibiting the molecular interaction between the CD163 and GP4 proteins (Figure 8E). In order to ensure the steric hindrance deeply, blocking ELISA was also used to evaluate the influence of mAb‐5F2 on the combination between GP4 and CD163. The results of the blocking ELISA revealed that mAb‐5F2 binding resulted in a significant decrease in GP4 binding to CD163, indicating that its binding epitope hindered the interaction between GP4 and CD163 (Figure 8F). In addition, mAb‐5F2 inhibited GP4‐CD163 binding in a dose‐dependent manner at different concentrations (0.1–100 µg mL^−1^), and the IC_50_ was 5.909 µg mL^−1^, indicating that mAb‐5F2 was highly effective in blocking the binding of GP4 to CD163 (Figure 8G). The interaction between GP4^Q48A^ or GP4^I50A^ and CD163 was determined. The results indicated that both the GP4^Q48A^ and GP4^I50A^ mutants maintained their ability to bind to CD163 (Figure 8H). These findings collectively suggest that mAb‐5F2 sterically blocks the interaction between GP4 and CD163 through binding to residues Q48 and I50, thereby impairing virus internalization.

### 2.8 Production of the Recombinant Porcine mAb 5F2‐pFc

**Figure 9:**
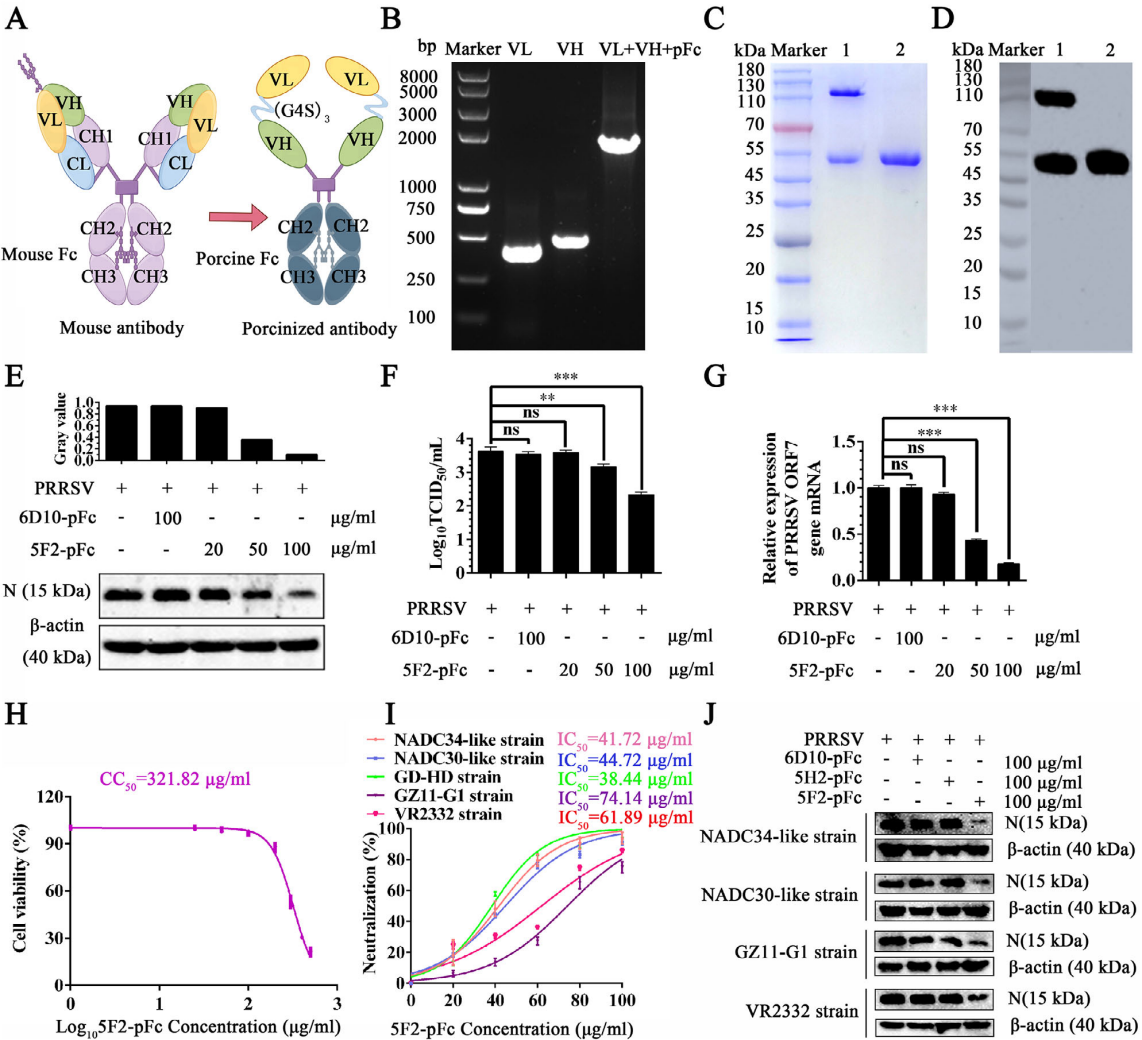
The recombinant porcine mAb 5F2‐pFc retained neutralization activity against PRRSV in PAMs. A) A schematic diagram illustrating the structure of the 5F2‐pFc variant of the recombinant porcine monoclonal antibody. B) The recombinant gene was inserted into the pLVX‐IRES‐ZsGreen vector. C) In vitro‐purified 5F2‐pFc dimerization was analyzed via SDS‐PAGE. D) In vitro purified 5F2‐pFc dimerization was analyzed via western blotting, and goat anti‐pig HRP was used as the detection antibody. Line 1: 5F2‐pFc under nonreducing conditions; line 2: 5F2‐pFc under β‐mercaptoethanol reduction conditions. E) The neutralizing activity of 5F2‐pFc was evaluated by western blotting analysis of PRRSV N protein levels, F) ORF7 mRNA levels, and G) the progeny virus titer. H) The CC_50_ value of 5F2‐pFc was determined via a CCK‐8 assay. I) The IC_50_ values of 5F2‐pFc against the NADC34‐like, NADC30‐like, GD‐HD, GZ11‐G1, and VR2332 strains were determined via immunofluorescence. J) The neutralizing activities of 5F2‐pFc were evaluated via western blotting analysis of PRRSV N protein levels. The symbols represent three independent biological replicates. The data were collected via Microsoft Excel (Excel 2016). Statistical analysis was performed via GraphPad Prism version 9.0 (GraphPad Software, San Diego, CA, USA). The data are presented as the means ± SD from a representative experiment. *p*‐values were calculated using one‐way analysis of variance (ANOVA), with Dunnett's or Tukey's multiple comparisons post hoc tests employed to assess statistical significance. Statistical significance is indicated as follows: *p* < 0.05 (^∗^), *p* < 0.01 (^∗∗^), *p* < 0.001 (^∗∗∗^), and “ns” denotes no significant difference.

To reduce the immunogenicity and enhance the safety profile of mAb‐5F2 for porcine applications, we engineered a recombinant porcine mAb 5F2‐pFc variant (**Figure** **9**A). The variable heavy (VH) and light (VL) chains of mAb‐5F2 were amplified, linked via a (G_4_S)_3_ linker, and fused with the porcine IgG1 Fc fragment (Figure 9B). A stable HEK293F cell line that secretes this recombinant antibody was subsequently established through lentiviral transduction. Purified 5F2‐pFc was analyzed by SDS‐PAGE and western blotting, revealing a distinct band corresponding to the expected ≈56 kDa molecular weight under the condition of β‐mercaptoethanol reduction (Figure 9C,D). Under nonreducing conditions, a clear band at 112 kDa was observed, indicating that it forms a homodimer through the disulfide bond between porcine IgG1 Fc fragments (Figure 9C,D). The final purification yield reached 500 mg L^−1^. Functional assays confirmed that 5F2‐pFc retained dose‐dependent neutralization activity against the PRRSV GD‐HD strain in PAMs, as evidenced by reduced PRRSV N protein expression (Figure 9E), decreased ORF7 mRNA levels (Figure 9F), and lower progeny virus titers (Figure 9G). To determine that the anti‐PRRSV activity of 5F2‐pFc cannot be attributed to its cytotoxic effect, the CC_50_ and IC_50_ values were determined in PAMs. The CC_50_ value of 5F2‐pFc was 321.82 µg mL^−1^ (Figure 9H). The neutralization potency was quantified, with an IC_50_ value of 38.44 µg mL^−1^ against the GD‐HD strain in PAMs (Figure 9I). Notably, 5F2‐pFc also effectively neutralized other strains: 41.72 µg mL^−1^ (NADC34‐like strain), 44.72 µg mL^−1^ (NADC30‐like strain), 61.89 µg mL^−1^ (VR2332 strain), and 74.14 µg mL^−1^ (GZ11‐G1 strain) (Figure 9I). Functional validation confirmed that 5F2‐pFc could effectively inhibit PRRSV replication in PAM cells. Compared with isotype‐matched controls 6D10‐pFc and 5H2‐pFc, treatment with 5F2‐pFc significantly reduced PRRSV N protein levels at a concentration of 100 µg mL^−1^ (Figure 9J). These results indicated that 5F2‐pFc had cross‐neutralization efficacies against different PRRSV isolates in vitro.

### 2.9 In Vivo Efficacy of the Porcine mAb 5F2‐pFc Against PRRSV Infection

**Figure 10:**
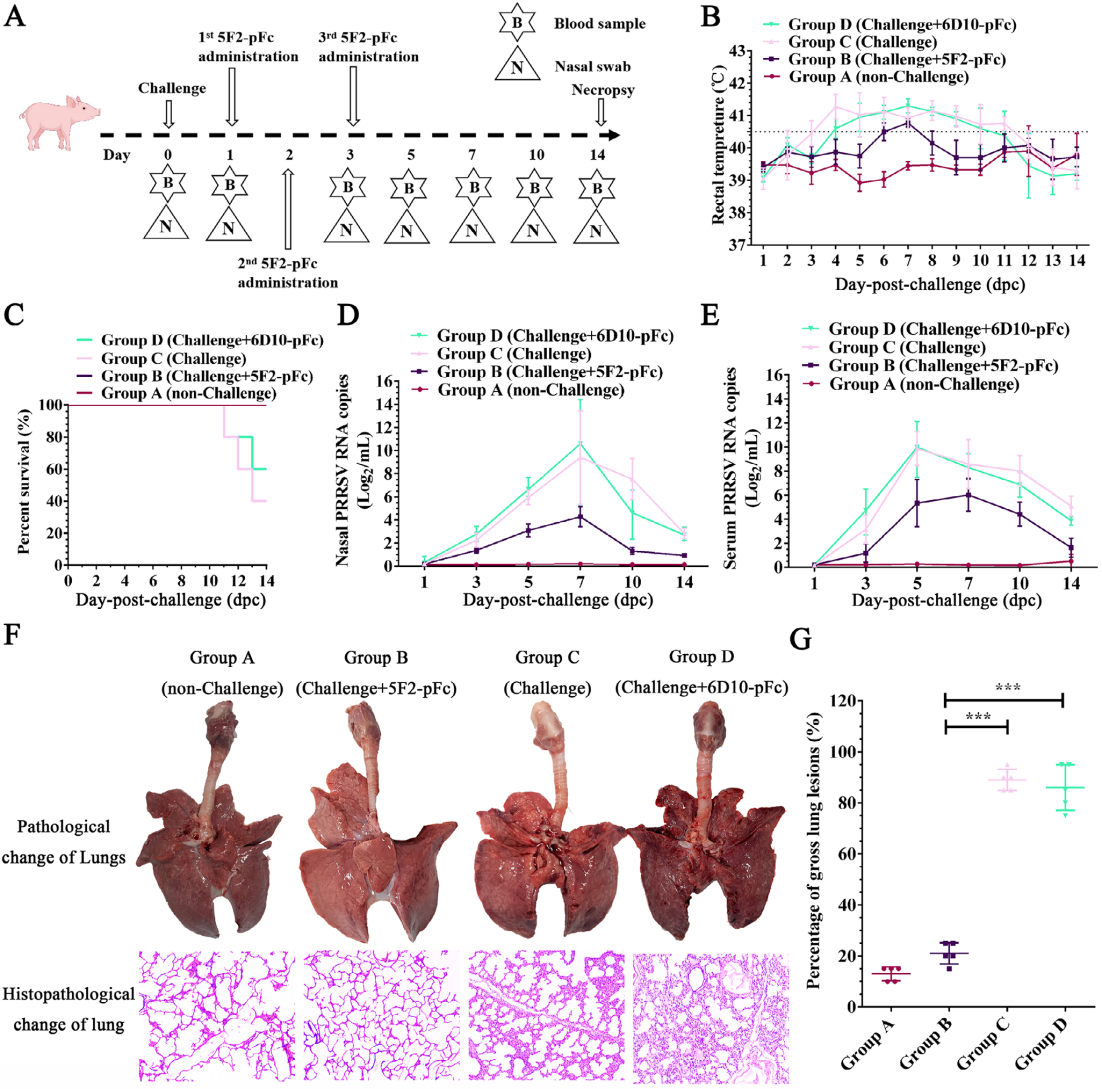
5F2‐pFc can inhibit PRRSV JXA1 strain replication in piglets and reduce the number of lung lesions. A) Schematic of the in vivo antiviral efficacy evaluation of 5F2‐pFc against PRRSV JXA1. B) Daily rectal temperature monitoring of surviving animals across treatment groups. C) Survival curves and mortality rates of piglets in each treatment group. D,E) qRT‐PCR quantification of PRRSV RNA in serum D) and nasal swabs E) Viremia analysis of different groups of pigs. F) Gross lung lesions (representative images captured postmortem at the time of death or 14 dpc) and histopathological analysis of H&E‐stained lung sections (scale bar: 100 µm). G) The scores of gross lung lesions in each piglet were based on the percentage of the lung area affected via a scoring (100‐point) system. Each lung lobe was assigned several points (100 points in total). The symbols represent three independent biological replicates. The data were collected via Microsoft Excel (Excel 2016). Statistical analysis was performed via GraphPad Prism version 9.0 (GraphPad Software, San Diego, CA, USA). *p*‐values were calculated using one‐way analysis of variance (ANOVA), with Dunnett's or Tukey's multiple comparisons post hoc tests employed to assess statistical significance. Statistical significance is indicated as follows: *p* < 0.05 (^∗^), *p* < 0.01 (^∗∗^), *p* < 0.001 (^∗∗∗^), and “ns” denotes no significant difference.

To evaluate the in vivo efficacy of the porcine mAb 5F2‐pFc, we conducted a controlled animal challenge experiment (**Figure** **10**A). To minimize animal use, we followed Jaykaran Charan's guidelines and used GPower to determine sample sizes, dividing the animals into four groups (*n* = 5).^[^ ^23^ ^]^ During the observation period, no redness or swelling was observed at the injection site of 5F2‐pFc, and no systemic adverse reactions were noted in the pigs. Rectal temperature monitoring revealed that pigs in Groups C and D presented elevated body temperatures (>40.5 °C) starting at 3 and 4 days post‐conception (dpc), respectively, which persisted through 11 dpc. In contrast, Group B pigs presented delayed and transient temperature elevation (>40.5 °C from 6 to 7 dpc), whereas Group A pigs remained normothermic throughout the study period (Figure 10B). Mortality occurred in Groups C and D beginning at 11 dpc, resulting in 40% and 60% survival rates by 14 dpc, respectively. Even if some pigs survive, the pig herd presents symptoms such as dyspnea, high fever, and anorexia. The production performance of these affected individuals is compromised, leading to their timely elimination. Notably, Group B maintained a 100% survival rate at this time point, which was consistent with the unchallenged group (Group A) (Figure 10C).

Quantitative RT‐PCR analysis of serum and nasal swabs revealed significantly lower PRRSV RNA levels in Group B than in Groups C and D, with no PRRSV replication in Group A (Figure 10D,E). Pathological evaluation at 14 dpc revealed severe hemorrhage and edema in the lungs of Groups C and D. In contrast, in Group B, only a few bleeding spots and mild edema were observed in two pigs, whereas in Group A, the pathological appearance of the lungs was normal (Figure 10F,G). Histopathological changes in the lungs revealed thickened alveolar walls, erythrocyte infiltration, and inflammatory cell accumulation in Groups C and D. In contrast, intact alveolar structures were observed in Groups A and B (Figure 10F).

A standardized gross lesion scoring system^[^ ^24^ ^]^ further quantified pulmonary damage, revealing significantly higher scores in Groups C (90.3 ± 4.9) and D (86.7 ± 9.4) than in Group B (21.5 ± 4.1; Figure 10G). Collectively, these results demonstrate that the intramuscular administration of 5F2‐pFc effectively enhances survival rates, reduces viral dissemination, and alleviates pulmonary pathology in PRRSV‐infected pigs. These findings suggest that 5F2‐pFc is a promising therapeutic candidate for controlling PRRSV, offering significant economic benefits to the swine industry through reduced disease burden and associated losses.

## 3 Discussion

Our study aimed to identify a broadly neutralizing antibody that targets PRRSV and elucidate its mechanism of action, with dual objectives of identifying epitopes for subunit vaccine development and establishing an antibody‐based antiviral therapy platform. Through systematic analysis, we determined that mAb‐5F2 recognized two conserved key amino acids (Q48 and I50) on GP4, which inhibited virus internalization by steric hindrance of the binding of GP4 to CD163. This discovery addresses critical challenges in the prevention and treatment of PRRSV.

Neutralizing epitopes serve as ideal targets for subunit vaccines because of their ability to elicit protective immunity without viral interference.^[^ ^25^ ^]^ Previous PRRSV vaccine strategies focused on envelope proteins (GP5, GP3, GP4, M), but their high variability limits cross‐strain protection.^[^ ^26^ ^]^ For example, GP5‐based vaccines targeting linear epitopes (e.g., aa 32–55, aa 73–93) have shown strain‐specific efficacy.^[^ ^27^ ^]^ To achieve this challenge, an innovative approach was employed in this study. Initially, the use of purified PRRSV as an immunogen results in better antigenicity and can more precisely imitate the natural infection state than traditional exogenously expressed protein antigens. However, the purified PRRSV particles were derived from PRRSV‐infected MARC‐145 cells and may have cross‐reacted with other cellular or nonspecific antigens (Figure 3C). During this process, we employed two ELISA methods to eliminate monoclonal antibodies (mAbs) that bind to the N protein (Figure 2B). We used PRRSV‐infected and uninfected MARC145 cells for IFA to eliminate the influence of other cellular or nonspecific antigens (Figure 2C). Fortunately, we successfully screened five monoclonal antibodies (mAbs) that specifically bind to PRRSV. We further identified two monoclonal antibodies, mAb‐5H2 and mAb‐5F2, which were obtained via in vitro neutralization evaluation. Although mAb‐5H2 showed a high neutralizing titer against the PRRSV GD‐HD strain (IC_50_ = 22.46 µg mL^−1^), it did not show any neutralizing activity against strains of other lineages (such as VR2332 and NADC30) (Figures 3 and 4). Therefore, mAb‐5H2 may recognize an epitope specific to the GD‐HD strain, whereas mAb‐5F2 is likely to target a broadly neutralizing epitope conserved across PRRSV strains. A more in‐depth characterization of the broadly neutralizing epitope is necessary to advance the development of effective strategies for preventing and controlling PRRSV infection.

Our study elucidates the crucial role of the interaction between GP4 (aa 44–70) and CD163 in the process of viral internalization. Previous studies have utilized phage panning technology to screen for peptides capable of blocking the interaction between CD163 and GP4, predicting that GP4 (aa 23–50) is the region involved in binding to the SRCR5 domain of CD163.^[^ ^22^ ^]^ However, this method has limitations in accurately identifying the precise binding regions, which may not represent the most critical interaction sites. In contrast, the present study employed a GP4 truncation approach to analyze its binding region with CD163 systematically. The results demonstrated that GP4 (aa 54–178) exhibited reduced binding affinity to CD163, whereas GP4 (aa 70–178) completely lost the ability to bind, suggesting that both the aa 44–54 and 54–70 regions affect the interaction between GP4 and CD163. In the future, the interaction between PRRSV and CD163 will be investigated using cryo‐electron microscopy.^[^ ^28^ ^]^ In addition, several studies have confirmed that CD163 serves as an indispensable receptor for both PRRSV uncoating and internalization.^[^ ^11^, ^29^ ^]^ In this study, we demonstrate that mAb‐5F2 does not affect PRRSV adsorption but does impair PRRSV internalization. Furthermore, we show that mAb‐5F2 sterically hinders CD163 binding to GP4, thereby inhibiting viral internalization. Since CD163 also functions as a primary receptor for PRRSV uncoating^[^ ^11^, ^29^ ^]^ future studies should determine whether mAb‐5F2 blocks this uncoating process.

Our study demonstrated that mAb‐5F2 recognizes the conformational epitope of GP4, specifically targeting two conserved key amino acids: Q48 on the α helix and I50 in the loop (Figure 7), which can sterically block the interaction between GP4 and the host receptor CD163. mAb‐5F2 inhibited the binding of GP4‐CD163 in a dose‐dependent manner at different concentrations (Figure 8).^[^ ^30^ ^]^ This finding aligns with recent efforts using self‐assembling nanoparticles expressing mixed GP2‐GP3‐GP4 antigens to reduce clinical symptoms and viral loads.^[^ ^31^ ^]^ Unlike these multiepitope approaches, mAb‐5F2's single‐epitope specificity offers advantages in minimizing immune interference while maintaining broad‐spectrum activity across diverse PRRSV strains (Figure 4). Previous studies have demonstrated that a novel strategy for an anti‐idiotype vaccine is the use of a monoclonal antibody that mimics the neutralization epitope of the PRRSV GP5 protein, and can provide immune protection to pigs.^[^ ^32^ ^]^ On this basis, we will immunize mice with mAb‐5F2 and screen for Ab2β anti‐idiotypic monoclonal antibodies with mirror structures of the neutralizing antigenic epitopes of PRRSV, which will be utilized for the prevention and control of PRRSV in the future.

mAb‐5F2 exerts its antiviral effect by blocking GP4‐CD163 binding at the stage of internalization (Figure 8A,B). This mechanism prevents viral entry into host cells, as evidenced by reduced PRRSV RNA levels (Figures 3, 4, and 5B), decreased progeny titers (Figures 3E and 4F), and preserved alveolar integrity in vivo (Figure 10F,G). Notably, the IC_50_ value of 38.44 µg mL^−1^ for 5F2‐pFc in PAMs (Figure 9I) indicates its potency. However, the neutralization activity of 5F2‐pFc was lower than that of mAb‐5F2, likely due to the direct linkage of the heavy and light chains via a (G_4_S)_3_ linker, which may have altered the antibody's structural conformation and consequently affected its functional activity. We verified the neutralization ability of 5F2‐pFc and mAb‐5F2 against all PRRSV isolates in vitro and systematically resolved the mechanisms underlying their broad‐spectrum neutralizing activity. Considering the characteristics of the isolates in lineages 1 and 8, the isolates in lineage 1 had lower pathogenicity than the ones in lineage 8. To evaluate the therapeutic effect of 5F2‐pFc more clearly, the highly pathogenic JXA1 strain (lineage 8) was selected for in vivo antiviral experiments. However, many studies have confirmed that some lineage 1 isolates can also lead to high mortality.^[^ ^12^, ^33^ ^]^ Therefore, the therapeutic efficacy of 5F2‐pFc against currently predominant lineage 1 isolates should be evaluated in the near future. In addition, the porcine format (IgG1 Fc) minimizes immunogenicity in pigs.^[^ ^22^ ^]^ Animal experiments revealed that 5F2‐pFc had a good therapeutic effect. In a preliminary experiment to evaluate the therapeutic effect of 5F2‐pFc, we initiated 5F2‐pFc treatment after the clinical symptoms were observed, and the results revealed that the viral load in the treatment group was significantly lower than that in the challenge group (data not shown). It showed a definite effect on PRRSV‐challenged pigs and confirmed the neutralizing effects of 5F2‐pFc in vivo. Moreover, the treatment delayed the death of the animals but did not result in a different survival rate. The potential explanation for the effect might be that PRRSV enters the stage of massive replication on day 3 of infection, making it difficult to completely inhibit viral proliferation with a single treatment. So, in the present study, the treatment time has been adjusted to be the 1st to 3rd day‐post‐challenge (dpc). The results revealed an increase in survival to 100% and a significant decrease in the viral load, indicating that 5F2‐pFc single treatment can provide 100% protection for infected pigs before the virus multiplies greatly. However, pigs still exhibited a transient fever and a low level of viremia in Group B (challenge + 5F2‐pFc) because PRRSV has not been completely eradicated (Figure 10B,D,E). The possible reason is that 5F2‐pFc treated at one time is not sufficient to completely eliminate PRRSV proliferation in pigs. Nevertheless, it was also confirmed that 5F2‐pFc had a therapeutic effect in pigs. These properties position 5F2‐pFc as a promising therapeutic candidate, particularly given the current lack of clinically approved treatments for PRRSV. The development of therapeutic drugs is a multifaceted endeavour—not limited to the injection of therapeutic antibodies. On the basis of these results, we will collaborate with multidisciplinary specialists to expedite the development of this antibody into a therapeutic drug through IND‐enabling development.

We established a stable HEK293F cell line expressing 5F2‐pFc at a yield of 500 mg L^−1^ (Figure 9A–D), leveraging its efficient protein secretion capacity for scalable production.^[^ ^34^ ^]^ This platform addresses the limitations of traditional hybridoma methods, which are plagued by low throughput and strain variability. The 5F2‐pFc platform demonstrates dual utility, serving as both a prophylactic vaccine adjuvant through subunit formulations and an antiviral therapeutic. Its ability to reduce viral loads in serum and pulmonary tissues (Figure 10D,E,G) suggests potential integration into combination therapies. Moreover, several researchers have demonstrated that FcγR cross‐linking induces cytokine production, which may have an inhibitory effect on PRRSV infection.^[^ ^35^ ^]^ Our findings complement existing work on PRRSV mAbs by introducing a broadly neutralizing candidate with defined mechanisms of action.

While this study advances the discovery of PRRSV antibodies, challenges remain. The hybridoma‐based approach has inherent inefficiencies, and low‐affinity mAb variants require optimization.^[^ ^36^ ^]^ By incorporating high‐throughput sequencing and machine learning, future studies could optimize antibody affinity and broaden epitope coverage.^[^ ^37^ ^]^ For example, predicting antibody conformations via GROMACS simulations^[^ ^36^ ^]^ or training models on GP4 polymorphism data^[^ ^38^ ^]^ may yield even more potent neutralizing agents. Future workflows may incorporate single‐cell B‐cell sorting^[^ ^38^ ^]^ or phage display libraries^[^ ^39^ ^]^ to isolate rare neutralizing clones. Additionally, validating 5F2‐pFc by challenge with different PRRSV strains and long‐term field trials will be essential for clinical adoption.

This research establishes mAb‐5F2 as a cornerstone for PRRSV control. By targeting a conserved epitope on GP4 and employing a porcine antibody platform, we created a versatile tool for antiviral therapy. These findings pave the way for next‐generation interventions to combat PRRSV, a major economic burden in swine production.

## 4 Experimental Section

### Cells and Viruses

SP2/0 myeloma cells were obtained from ATCC and cultured in RPMI 1640 medium (Biological Industries, Israel) supplemented with 20% fetal bovine serum (FBS). Monkey embryonic kidney epithelial cells (MARC‐145) were similarly maintained in Dulbecco's modified Eagle's medium (DMEM; Thermo Fisher Scientific, Waltham, MA, USA) supplemented with 10% FBS. Porcine alveolar macrophages (PAMs) were isolated from 4‐week‐old PRRSV‐negative pigs and propagated in RPMI 1640 medium supplemented with 10% FBS, as previously described.^[^ ^40^ ^]^ The study employed PRRSV strains, including the GD‐HD strain (GenBank: KP793736.1, PRRSV‐2 lineage 8), JXA1 strain (GenBank: EF112445.1, PRRSV‐2 lineage 8), NADC30‐like strain (GenBank: KX766379, PRRSV‐2 lineage 1), VR2332 strain (GenBank: EF536003.1, PRRSV‐2 lineage 5), and GZ11‐G1 strain (GenBank: KF001144.1, PRRSV‐1).

### Preparation of PRRSV Particles

The GD‐HD strain of PRRSV was propagated in MARC‐145 cells to generate 1 L of viral culture supernatant. This supernatant was concentrated to 10 mL via ultrafiltration. Following centrifugation at 8000 × g for 30 min to clarify the lysate, the clarified supernatant was subjected to high‐speed centrifugation at 20000 × g for 2.5 h to pellet virions. The resulting pellet was resuspended in 10 mL of phosphate‐buffered saline (PBS, pH 7.4). A 5–8 mL aliquot of virus‐containing supernatant was transferred to an ultracentrifuge tube and overlaid sequentially with 25%, 35%, 45% and 55% sucrose gradients. After ultracentrifugation at 110 000 × g for 2.5 h, distinct virus bands were observed between the 25–35%, 35–45% and 45–55% sucrose layers. These bands were carefully collected via a long needle and distributed into individual containers. A final centrifugation at 110 000 × g for 2 h was performed to remove residual sucrose, after which the pelleted virus was resuspended in 2 mL of PBS for downstream applications.

### Immunization of BALB/c Mice

The animal experiments were conducted in accordance with the guidelines of the Northwest A&F University Institutional Committee for the Care and Use of Laboratory Animals and were approved by the Committee on the Ethical Use of Animals of Northwest A&F University (approval number: DY2023078). Five BALB/c mice were subcutaneously immunized with 300 µg of purified intact PRRSV particles emulsified in Freund's complete adjuvant. Two booster vaccinations were administered intramuscularly via 300 µg of viral particles in incomplete Freund's adjuvant at 2‐week intervals. A final intravenous booster immunization (300 µg of PRRSV particles via tail vein injection) was performed 1 week prior to splenocyte collection. Splenocytes were isolated from the spleens of immunized mice and fused with SP2/0 myeloma cells via a polyethylene glycol (PEG)‐mediated fusion protocol.

### Screening of Monoclonal Antibodies

Spleen cells were isolated from mice one week postshock immunization and fused with SP2/0 myeloma cells via polyethylene glycol 1500 (mIbio, Cat. No. 25322‐68‐3). The hybridoma cultures were maintained in RPMI 1640 medium (supplemented with 10% FBS; Gibco, Carlsbad, CA, USA) containing hypoxanthine‐aminopterin‐thymidine (HAT). Negative selection for PRRSV N protein reactivity and positive screening for virus particle recognition were performed via indirect ELISA. ELISA‐positive clones were further validated through indirect immunofluorescence assays (IFAs) on PRRSV‐infected and PRRSV‐uninfected MARC‐145 cells. Neutralizing antibody activity was confirmed via virus neutralization assays. The selected hybridomas were subjected to three rounds of limiting dilution cloning to establish monoclonal cell lines. Next, the mAb was purified from the supernatants of different hybridomas via protein G Sepharose beads (Amersham Bioscience, Shanghai, China). Finally, the purified mAb was analyzed by SDS‐PAGE and quantified using a BCA protein assay kit (TaKaRa, Dalian, China).

### Indirect ELISA

Since PRRSV particles have the highest abundance of N protein^[^ ^41^ ^]^ and extensive studies have demonstrated that anti‐PRRSV N protein antibodies lack neutralizing activity against PRRSV infection, we employed two ELISA strategies to screen for mAbs specific to PRRSV particles but not cross‐reactive with the N protein. For this purpose, 96‐well microplates were separately coated with PRRSV N protein (200 ng per well) and PRRSV particles (200 ng per well) in carbonate buffer (pH 9.6) overnight at 4 °C. The recombinant N protein used in this study was produced and purified as previously described.^[^ ^41^ ^]^ After coating, the plates were washed three times with PBS‐T (0.05% Tween 20) and blocked with 2.5% skim milk in PBS‐T for 1 h at 37 °C. Following three additional washes, hybridoma supernatants (100 µL per well), PRRSV‐immunized mouse serum, and unimmunized mouse serum were added to the respective wells and incubated for 1 h at 37 °C. Three subsequent washes were performed before the addition of an HRP‐conjugated goat anti‐mouse IgG antibody (1:5000 dilution; 100 µL per well) in blocking buffer for 1 h at 37 °C. After the final wash, 50 µL of TMB substrate solution (TianGen Biotech, Beijing, China) was added to each well, and the mixture was incubated at room temperature for 15 min. The reaction was terminated by adding 50 µL of 3 m H_2_SO_4_, and the optical density (OD) values were immediately measured at 450 nm via a Multiskan SkyHigh Microplate Spectrophotometer.

To precisely identify the epitopes recognized by 5F2, affinity analysis was performed using an ELISA method. Taking GP4 protein as an example, 96‐well microplates were coated with varying concentrations of GP4, blocked with 2.5% skim milk in PBS‐T, and washed three times with PBS‐T. Each well was then incubated with 100 µL of mAb‐5F2 at 10 µg mL‐1. A goat anti‐mouse HRP‐conjugated antibody (1:500 dilution) was used as the secondary antibody, and the OD450 value was measured after color development. All experiments were performed in triplicate, with three separate experimental wells used for each repetition. Finally, the data were statistically collected using Excel, visualized using Grouppad, and the Bmax and Kd values were calculated.

### Indirect Immunofluorescence Assay (IFA)

To determine whether hybridoma supernatants bind to PRRSV‐infected MARC‐145 cells, we performed IFA as previously described.^[^ ^39^ ^]^ MARC‐145 cells were infected with the PRRSV‐2 GD‐HD strain at an MOI of 0.1. Then, at 36 h, the PRRSV‐2‐infected and uninfected MARC145 cells were fixed with 4% paraformaldehyde at 4 °C for 30 min. Following two washes with PBS, the cells were blocked with 1% bovine serum albumin (BSA) in PBS for 1 h at 37 °C. After three additional washes, the cells were incubated with hybridoma supernatants (100 µL per well) at 37 °C for 1 h. The cells were subsequently washed again and labeled with Alexa Fluor 488‐conjugated goat anti‐mouse IgG (H&L chain specific, 1:500 dilution; Thermo Fisher Scientific) for 1 h at 37 °C. Simultaneously, the nuclei were stained with DAPI dihydrochloride (Beyotime, C1006) at 25 °C for 15 min. Finally, the cells were visualized via an inverted fluorescence microscope (Leica AF6000, Germany) with appropriate filter sets. All experiments were performed in triplicate and repeated at least three times.

### Identification of mAb Subtypes

The isotype distribution of the mAbs was determined via the mAb Subtype Identification Kit (Baililab Biology, Beijing, China) according to the manufacturer's instructions. Hybridoma supernatants (diluted 1:100 in PBS) were added to the wells (50 µL per well), followed by the addition of HRP‐conjugated goat anti‐mouse IgA/IgM/IgG antibodies (1:200 dilution; 50 µL per well). The plate was sealed with adhesive film and incubated at room temperature for 1 h. Following incubation, a TMB substrate solution (100 µL per well) was added, and the mixture was incubated in the dark for 10 min at room temperature. The reaction was terminated by adding a stop solution (100 µL per well), and the OD_450 nm_ values were measured using a Multiskan SkyHigh Microplate Spectrophotometer.

### Cell Viability Analysis

To analyze the cytotoxicity of the mAbs and recombinant porcine neutralizing mAb, cell viability was evaluated via a CCK‐8 assay (Beyotime Institute of Biotechnology, Beijing, China) according to the manufacturer's instructions. Briefly, PAMs were cultured in 96‐well plates at a density of 1 × 10 ^^5^ cells were incubated for 6 h and then treated with different concentrations of mAbs or recombinant porcine neutralizing mAbs (0–500 µg mL) in RPMI 1640 supplemented with 3% FBS for 24 h. Then, the CCK‐8 reagent (10 µL per well) was added, and the samples were incubated in 5% CO_2_ at 37 °C for 1 h. The OD_450nm_ values were measured using a Multiskan SkyHigh Microplate Spectrophotometer to determine cell viability. All experiments were performed in triplicate, with three separate experimental wells used for each repetition. The data are presented as the means ± SD from a representative experiment. Finally, the data were statistically collected using Excel, visualized using Grouppad, and the CC_50_ was calculated.

### Neutralization Assay

For virus neutralization analysis, PAMs and MARC‐145 cells were cultured in 24‐well plates. Each experimental group included three biological replicates, with triplicate wells pooled for RNA extraction. Negative controls (PRRSV only) and irrelevant monoclonal antibody (mAb) controls were maintained throughout the experiment. Diluted sera (50 µg mL) from immunized mice or mAbs were mixed with PRRSV strains (the VR2332 strain, GZ11‐G1 strain, NADC30‐like strain, NADC34‐like strain, and GD‐HD strain; 0.1 MOI) in RPMI 1640/DMEM at 37 °C for 1 h. The antibody‐virus mixtures were then added to the cells and incubated at 37 °C in 5% CO_2_ for 2 h. After being washed, the cells were replenished with RPMI 1640/DMEM containing 3% FBS and further incubated at 37 °C in 5% CO_2_ for 24 h. Viral infection and replication were assessed by quantifying the intracellular PRRSV N protein via western blotting with the anti‐N mAb‐6D10,^[^ ^15^ ^]^ measuring PRRSV RNA copies via RT‐qPCR,^[^ ^15^ ^]^ and TCID_50_ analysis of the progeny virus. Total RNA was extracted using the Qiagen RNeasy Mini Kit and then reverse‐transcribed with TaKaRa PrimeScript RT Master Mix. RT‐qPCR was performed on the StepOnePlus System (Applied Biosystems, Thermo Fisher Scientific) with RealStar Green Fast MasterMix (Roche) and ORF7‐specific primers, with β‐Tubulin used as an internal control, β‐Tubulin‐F (GAACATGGCCGAGATCGTC), β‐Tubulin‐R (GTCGCAGTTGTTACCAACTGT), ORF7‐F (AAACCAGTCCAGAGGCAAGG), and ORF7‐R (GCAAACTAAACTCCACAGTGTAA). Finally, viral titers in the cell supernatants were determined by TCID_50_ analysis in MARC‐145 cells via the Reed‐Muench method.^[^ ^15^ ^]^ Additionally, to screen neutralizing mAbs, the mAbs and recombinant HP‐PRRSV HuN4‐EGFP strains were incubated at a multiplicity of infection (MOI) of 0.1 at 37 °C for 1 h. Finally, to analyze viral infection and replication, a flow cytometer was used to obtain the percentage of infected cells.

To evaluate the dose dependence of the selected mAbs with neutralization activity, serial dilutions of mAbs (0–50 µg mL^−1^) were preincubated with the GD‐HD strain (0.1 MOI) at 37 °C for 1 h before cell addition. Western blotting, RT‐qPCR, and TCID_50_ analysis of progeny viruses were used to evaluate the neutralization activity of the mAbs at different doses.

To evaluate the neutralizing activity of the recombinant neutralizing mAb against different PRRSV isolates, we also performed western blotting, RT‐PCR, and TCID_50_ analysis as described above. Additionally, standardized IC_50_ determination was employed to assess the neutralizing activity of the recombinant porcine neutralizing mAb against various PRRSV isolates.

All experiments were performed in triplicate, with three separate experimental wells used for each repetition. Finally, the line chart displays the mean ± SD values of the samples, along with *P* values as indicated, which were calculated using one‐way ANOVA with Dunnett's multiple‐comparison test.

### Western Blotting

To evaluate the neutralizing activity of the mAbs against PRRSV and their dose‐dependent neutralization abilities against different PRRSV strains, we performed western blotting analysis as previously described.^[^ ^12b^ ^]^ Equal protein amounts from cell lysates were subjected to SDS‐PAGE separation and subsequently transferred to nitrocellulose membranes. The membranes were blocked with 5% bovine skim milk in PBS‐T for 1 h at room temperature, followed by overnight incubation with mAb supernatants. After three washes with PBS‐T, the blots were probed with HRP‐conjugated goat anti‐mouse IgG (H+L chain specific, 1:6000; Proteintech, SA00001‐1) for 1 h at room temperature. Signal detection was achieved via an enhanced chemiluminescence (ECL) Plus Kit (Shanghai Jinpan Biotech Co., Ltd., Shanghai, China), and signals were visualized via the Tanon 5200 Chemiluminescent Imaging System. All immunoblots had three biological replicates, and the data were processed and visualized using Image Lab and GraphPad Prism.

### The 50% Inhibition Concentration (IC_50_) Assay

To evaluate the neutralizing activity of mAbs and recombinant porcine neutralizing mAbs against different PRRSV isolates, we performed a standardized IC_50_ determination as previously described.^[^ ^41^ ^]^ PRRSV (0.1 MOI) was preincubated with serially diluted mAbs (0–100 µg mL^−1^) in equal volumes at 37 °C for 1 h. The antibody‐virus complexes were then inoculated into MARC‐145 cells, which were subsequently cultured at 37 °C with 5% CO_2_ for 72 h. Viral infection was quantified via immunofluorescence detection of the PRRSV N protein in eight replicate wells per dilution. The data were collected via ImageJ software (https://imagej.nih.gov/ij/) and analyzed with GraphPad Prism 5.0 to determine the IC_50_ values. Each experiment was repeated three times to ensure experimental reproducibility.

### Attachment and Internalization Assays

The PRRSV attachment assay was conducted as previously described.^[^ ^41^ ^]^ PAMs were seeded into 24‐well plates and incubated at 37 °C for 6 h. The PRRSV GD‐HD strain (MOI of 5) was preincubated with neutralizing monoclonal antibodies (100 µg mL^−1^) at 37 °C for 1 h before being cooled to 4 °C. The cooled virus‐antibody complexes were then added to the PAMs and incubated at 4 °C for 1 h. Unbound virions were removed by washing the cells three times with cold PBS. Finally, PAMs were harvested for RT‐qPCR analysis of viral RNA levels and IFA quantification of surface‐bound virions.

For the internalization assay, PAMs were similarly seeded and preconditioned as described above. Following a 1‐h incubation with cooled virus‐antibody complexes at 4 °C, the cells were transferred to RPMI 1640 medium supplemented with 3% FBS and incubated at 37 °C for 30 min to allow for viral internalization. To terminate internalization, proteinase K (50 µg mL^−1^) was added to digest the extracellular virions at 4 °C for 45 min. After the enzymes were removed by washing, the PAMs were treated with PMSF to neutralize the activity of proteinase K. The cells were then harvested for RT‐qPCR and IFA to evaluate the intracellular viral load.

All experiments were performed in triplicate, with three separate experimental wells used for each repetition. The data are presented as the means ± SD from a representative experiment. Finally, the data were visualized using GraphPad Prism, with P values as indicated, and analyzed using one‐way ANOVA with Dunnett's multiple‐comparison test.

### Sandwich ELISA

To evaluate the ability of neutralizing mAbs to capture intact PRRSV particles, a sandwich ELISA was performed. The capture antibody (400 ng per well) was immobilized by overnight incubation in carbonate buffer (pH 9.6) at 4 °C. After being blocked with 5% skim milk in PBS‐T for 1 h at 37 °C, the wells were incubated with PRRSV particles (400 µL per well) at 37 °C for 1 h. The subsequent detection phase utilized nanobody Nb1‐HRP (targeting PRRSV N protein)^[^ ^20^ ^]^ diluted in blocking buffer. After a 1‐h incubation at 37 °C, the reaction was developed by adding 50 µL of TMB substrate and incubating at room temperature for 15 min. The enzymatic reaction was terminated with 50 µL of 3 m H_2_SO_4_, and the OD_450nm_ values were measured using a Multiskan SkyHigh Microplate Spectrophotometer. All experiments were performed in triplicate and repeated at least three times.

### Epitope Mapping of Neutralizing mAb

To identify the PRRSV structural protein recognized by the neutralizing mAb, we expressed six viral proteins (GP2, GP3, GP4, GP5, M, and N) in HEK293T cells via plasmids encoding HA‐tagged versions (pCAGEN‐GP2‐HA, ‐GP3‐HA, ‐GP4‐HA, ‐GP5‐HA, ‐M‐HA, and ‐N‐HA) as previously described.^[^ ^42^ ^]^ The transfected cells at 36 h post‐transfection (hpt) were analyzed via IFA. The cells were fixed with 4% paraformaldehyde, incubated with the neutralizing monoclonal antibody (mAb), and visualized via Alexa Fluor 488‐conjugated goat anti‐mouse IgG (H&L chain specific) under a fluorescence microscope.

To fine‐map the antigenic determinants, overlapping peptide fragments and point mutants of the identified structural protein were generated and expressed in HEK293T cells. IFA was repeated via the same protocol with the neutralizing mAb as the primary antibody to identify specific epitope regions. All experiments were performed in triplicate and repeated at least three times.

### Coimmunoprecipitation (Co‐IP)

Prior studies have established that the host factor CD163 serves as a primary receptor for PRRSV attachment and internalization.^[^ ^21^, ^30^ ^]^ To investigate whether the neutralizing mAb could block the interaction between PRRSV structural proteins and CD163, we performed Co‐IP as previously described.^[^ ^25b^ ^]^ HEK293T cells were individually transfected with the recombinant plasmids pCAGEN‐HA‐CD163‐myc and pCAGEN‐His‐GP4‐HA. At 72 hpt, the cells were lysed in 1% Nonidet P‐40 buffer [50 mm Tris‐HCl (pH 7.4), 150 mm NaCl, 1 mm PMSF, 2.5 µM TSA, and 25 mm NAM] containing protease inhibitors. The cell lysates were incubated overnight with the neutralizing monoclonal antibody (mAb) or mouse anti‐His tag antibody (1:500 dilution) at 4 °C. The protein complexes were subsequently immunoprecipitated via protein A/G agarose beads (Beyotime Institute of Biotechnology, Beijing, China) for 4 h, followed by three washes with PBS‐T. After the unbound proteins were removed, the precipitated samples were resolved via western blotting analysis to examine protein interactions.

To determine the specific domain through which GP4 interacts with CD163. HEK293T cells were individually transfected with the recombinant plasmids pCAGEN‐HA‐CD163‐myc, pCAGEN‐His‐GP4‐HA, ‐GP4^34‐178^‐HA, ‐GP4^44‐178^‐HA, ‐GP4^54‐178^‐HA, and ‐GP4^70‐178^‐HA. At 72 hpt, the cells were lysed in 1% Nonidet P‐40 buffer. Next, the mouse anti‐His tag antibody and protein A/G agarose beads were incubated together for 12 h at 4 °C. The mouse anti‐His beads were subsequently washed three times with PBS, and the cell lysates (from HEK293T cells individually transfected with the recombinant plasmids pCAGEN‐His‐GP4‐HA, ‐GP434‐178‐HA, ‐GP444‐178‐HA, ‐GP454‐178‐HA, and ‐GP470‐178‐HA) were incubated with the beads at 4 °C for 12 h. The protein‐bound beads were subsequently washed three times with PBS again. The cell lysates from pCAGEN‐HA‐CD163‐myc‐transfected HEK293T cells were subsequently incubated with the beads at 4 °C for 12 h and washed three times with PBS‐T. Finally, the immunoprecipitated proteins were analyzed via western blotting using rabbit anti‐HA as the primary antibody and goat anti‐rabbit‐HRP as the secondary antibody to investigate protein interactions. All experiments were performed in triplicate and repeated at least three times.

### Blocking ELISA (bELISA)

To analyze whether mAb‐5F2 binds to GP4 and thus blocks its binding to CD163, bELISA was employed to elucidate the underlying mechanism. First, ELISA plates were coated with GP4 (400 ng per well) in PBS at 4 °C overnight. Second, after being washed three times with PBS‐T, the cells were blocked with blocking buffer (200 µL per well) for 1 h at room temperature. Simultaneously, 100 µL of mAb‐5F2 (at various concentrations ranging from 0.1 to 100 µg mL^−1^) was incubated for 30 min at 37 °C. After washing three times with PBS‐T, 100 µl of serially diluted CD163 (10 µg Per well) was added for 30 min at 37 °C. After being washed three times with PBS‐T, the ELISA plates were incubated with mouse anti‐myc (1:5000 dilution) for 30 min at 37 °C. Then, the goat anti‐rabbit HRP antibody was added to the culture plate and incubated for 30 min at 37 °C. Finally, 3 M H_2_SO_4_ (50 µL per well) was used to stop the colorimetric reaction, and the OD_450nm_ values were read using an automated microplate reader. The data were collected via Microsoft Excel (Excel 2016) and visualized via GraphPad Prism.

### Homology Modeling and Molecular Docking

Following identification of the antigenic domains recognized by the neutralizing mAb, homology‐based 3D structure predictions were performed for the PRRSV structural proteins (GP2, GP3, and GP4), the neutralizing mAb (amino acid sequences were obtained from hybridoma cell sequencing), and the receptor CD163 via the AlphaFold 3 server (https://golgi.sandbox.google.com/). Subsequently, protein‐protein docking models were established via ZDOCK SERVER, and interaction sites were analyzed via the Swiss Model and Discovery Studio Client software. Then, visual analysis was conducted with PyMOL. Finally, to evaluate epitope conservation, amino acid sequence alignments of the identified epitopes across 15 PRRSV isolates from GenBank were conducted via the MegAlign program (Lasergene 7.2).

### Construction of HEK293F Cell Lines for Stable Expression of Recombinant Porcine‐Neutralizing mAb

To mitigate immune rejection in pigs and improve safety profiles, we established a HEK293F cell line stably expressing a recombinant porcine neutralizing monoclonal antibody (mAb) via a lentiviral vector system, as previously described.^[^ ^41^ ^]^ Briefly, total RNA was extracted from hybridoma cells, followed by reverse transcription to synthesize first‐strand cDNA. The variable light (VL) and heavy (VH) chain genes of the neutralizing mAb were amplified via nested PCR and PCR primers for cloning variable genes of rearranged immunoglobulin light and heavy chains (5′ primers for VL: GGGGATATCCACCATGGATTTTCAAGTGCAGATTTTCAG; 3′ primers for VL: GGATACAGTTGGTGCAGTCGACTTACGTTTKATTTCCARCTT; 5′ primers for VH: GGGGATATCCACCATGRACTTCGGGYTGAGCTKGGTTTT; 3′ primers for VH: GACHGATGGGGSTGTYGTGCTAGCTGNRGAGACDGTGA). The VL and VH genes of the mAb were subsequently linked with a (G_4_S)_3_ linker and then fused with the porcine IgG Fc fragment to construct the chimeric antibody. This recombinant gene was inserted into the pLVX‐IRES‐ZsGreen vector. For lentivirus production, HEK293T cells were cotransfected with pLVX‐IRES‐ZsGreen, psPAX2, and pMD2. G plasmids via HP DNA transfection reagent (GenScript, Nanjing, China) according to the manufacturer's protocol. At 72 h postinfection, the fluorescently labeled lentivirus particles were observed under a microscope, and the supernatants were collected. Recombinant HEK293F cells were transduced with the lentiviral supernatant, and green fluorescent cells were isolated via a high‐speed flow cytometer (TaKaRa, Dalian, China). The sorted green‐fluorescent HEK293F cells were subsequently cultured and sorted again via high‐speed flow cytometry. This operation was repeated until the green‐fluorescence HEK293F cell occupancy rate reached 99.99%. The resulting stable cell line was subsequently cultured in serum‐free medium, after which the recombinant porcine neutralizing monoclonal antibody (mAb) was secreted into the culture medium. The next day, 10% culture medium was added, and the culture was continued until the sixth day or until the cell viability decreased to 50%. The supernatant was collected and purified via protein G Sepharose beads (Amersham Bioscience, Shanghai, China). The purified recombinant porcine neutralizing mAb was analyzed by SDS‐PAGE and western blotting under both reducing and nonreducing conditions, using β‐mercaptoethanol. Finally, the purified neutralizing mAb was quantified via a BCA protein assay kit (TaKaRa, Dalian, China).

### Animal Experiments

The animal experiments were conducted in accordance with the guidelines of the Northwest A&F University Institutional Committee for the Care and Use of Laboratory Animals and were approved by the Committee on the Ethical Use of Animals of Northwest A&F University (approval number: DY2025045). To minimize animal use, we followed Jaykaran Charan's guidelines and used GPower to determine sample sizes.^[^ ^23^ ^]^ Twenty healthy 4‐week‐old piglets negative for PRRSV antibodies and RNA were randomly assigned to four experimental groups: Group A (unchallenged control), Group B (JXA1‐challenged + recombinant mAb‐treated), Group C (JXA1‐challenged only), and Group D (JXA1‐challenged + irrelevant mAb‐treated). The animals were housed under isolated conditions as described in Figure 10A. Groups B, C, and D were intramuscularly inoculated with 1 × 10⁵ TCID_50_ of the PRRSV JXA1 strain, whereas Group A received an equivalent volume of sterile PBS. At 1 day post‐challenge (dpc), Group B pigs were administered 10 mg (5F2‐pFc concentrations of 10 mg mL^−1^, 2 mg kg^−1^ for a 5 kg pig) of recombinant mAb intramuscularly, and Group D pigs received 10 mg of irrelevant control mAb via the same route.^[^ ^12b^ ^]^ Groups A and C continued to receive sterile PBS. This treatment schedule was repeated at 2 and 3 dpc. Nasal swabs and serum samples were collected at 0, 1, 3, 5, 7, 10, and 14 dpc, with daily monitoring of body temperature. Viral RNA levels in sera and nasal swabs were quantified by RT‐qPCR as previously described.^[^ ^12b^ ^]^ At 14 dpc, all the piglets were euthanized for gross lesion evaluation. Lung tissues were fixed in 4% paraformaldehyde and subjected to histopathological analysis via hematoxylin and eosin (H&E) staining. All the samples were tested in three independent biological replicates. All data were analyzed visually using GraphPad Prism and further analyzed via one‐way ANOVA with Dunnett's multiple comparison test, with *p* values indicated. Survival curves were analyzed for *p* values via the log‐rank (Mantel‐Cox) test.

### Statistical Analysis

All the results were derived from three independent experiments. The data are presented as the means ± SD from a representative experiment. The data were collected and analyzed via Microsoft Excel (Excel 2016) and visualized via GraphPad Prism. For comparisons involving more than two groups or two independent variables, one‐way analysis of variance (ANOVA) with Dunnett's or Tukey's multiple comparisons post hoc tests was employed to assess statistical significance. Differences between groups were evaluated using Student's two‐tailed *t*‐test. Statistical significance is indicated as follows: *p* < 0.05 (^∗^), *p* < 0.01 (^∗∗^), *p* < 0.001 (^∗∗∗^), and “ns” denotes no significant difference. Finally, all the figures were assembled via Adobe Photoshop CS5.1.

## Conflict of Interest

The authors declare no conflict of interest.

## Author Contributions

X.C. and J.Z. contributed equally to this work. Q.Z. performed conceptualization. X.C. and D.J. performed data curation. X.C. L.Z., and D.J. performed the formal analysis. X.C. and J.Z. performed the investigation. X.C., J.Z., P.J., X.L., H.N., Q.Z., X.L., J.S., and Y.S. performed the methodology. X.C., J.Z., and P.J. performed the project. X.C., J.Z., P.J., X.L., J.H., J.S., and Y.S. performed the resources. X.C., J.Z., P.J., H.N., and J.H. performed the software. X.C., J.Z., Q.Z., X.L., and Y.S. performed supervision. X.C., J.Z., H.N., L.Z., and Y.S. performed the validation. X.C. performed visualization. X.C., Y.S., and Q.Z. wrote the original draft and wrote, reviewed, and edited. Q.Z. performed funding acquisition. All the authors approved the final version of the manuscript.

## Data Availability

Data sharing is not applicable to this article as no new data were created or analyzed in this study.
